# Microfocused Ultrasound With Visualization (MFU-V) Effectiveness and Safety: A Systematic Review and Meta-Analysis

**DOI:** 10.1093/asj/sjae228

**Published:** 2024-11-14

**Authors:** Mojgan Amiri, Guardmond Ajasllari, Adea Llane, Gabriela Casabona, Tatjana Pavicic, Julia Sevi, Julieta Spada, Vasanop Vachiramon, Rossana Vasconcelos, Siew Tuck Wah, Taulant Muka, Sabrina Guillen Fabi

## Abstract

Microfocused ultrasound with visualization (MFU-V) is an advanced, noninvasive cosmetic procedure widely performed for skin lifting and tightening. We performed a systematic review and meta-analysis to evaluate the aesthetic effectiveness, patient satisfaction, skin quality, and safety profile of MFU-V treatment. A comprehensive search of 5 bibliographic databases up to 2023 was conducted. Pooled effect estimates with random effects models and corresponding 95% confidence intervals were calculated. Out of 4019 references, 42 studies were included. Meta-analysis showed 89% of patients (95% CI: 81%-94%; I^2^: 63%, *n* = 411) demonstrated some degree of global aesthetic improvement, as assessed by investigators. Similarly, 84% of patients (95% CI: 73%-91%; I^2^: 64%, *n* = 312) reported improvement following treatment. Satisfaction of any level was reported by 84% of patients (95% CI: 61%-94%; I²: 52%, *n* = 326), and 62% (95% CI: 37%-82%; I²: 3%, *n* = 172) when “neutral” as a response option was provided for patients. Skin quality (eg, wrinkles, texture) also improved. Patients reported a pooled mean pain score of 4.85 (95% CI: 4.35, 5.35; I^2^: 97%, *n* = 785), indicating moderate pain. Common adverse events included erythema, edema, swelling, bruising, and tenderness, all of which were generally mild to moderate in severity. Overall, our analysis demonstrated a notable increase in global aesthetic improvement and patient satisfaction following MFU-V treatment, accompanied by moderate pain and a generally favorable safety profile. However, the potential misclassification of neutral responses as positive may result in an overestimation of the treatment's efficacy. These findings highlight the need for well-designed trials to further explore MFU-V's clinical applications.

**Level of Evidence: 3 (Therapeutic):**

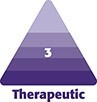

Microfocused ultrasound with visualization (MFU-V), an energy-based device, is widely recognized as one of the most effective and clinically relevant aesthetic treatment options for nonsurgical lifting and skin tightening.^1,2^ MFU-V has received clearance from the US Food and Drug Administration as a noninvasive procedure designed to elevate the brow, submental, and neck tissue and reduce wrinkles on the upper chest.^3^ The visualization feature of MFU-V facilitates precise and accurate delivery of energy by enabling real-time imaging of tissue layers.

Skin damage and age-related changes affect the aesthetic appearance and skin quality parameters.^4^ Studies have reported on the aesthetic effectiveness of MFU-V and its effects across a spectrum of aesthetic outcomes, patient satisfaction levels, and skin quality parameters such as skin firmness, surface, and tone evenness.^5-12^ Although these studies generally indicate MFU-V to be efficacious, there exists variability in the levels of improvement and satisfaction reported by patients and clinicians, ranging from high to moderate.^6-8,10,13,14^ Various factors may contribute to these discrepancies, including the energy level utilized, density and distribution of energy administration, duration of follow-up, and characteristics of the patients, yet these factors remain largely unexplored.

In the current comprehensive study, we systematically synthesized the existing evidence pertaining to the aesthetic effectiveness, skin quality, patient satisfaction, and safety associated with MFU-V treatment to provide a nuanced understanding of its implications. Our study describes the findings and limitations and identifies research gaps in the current literature. Furthermore, we quantitatively assessed the evidence, specifically focusing on the patient and investigator overall aesthetic improvement assessment, patient satisfaction, and pain scores following MFU-V.

## METHODS

This systematic review adhered to recent systematic review guidelines and the PRISMA reporting standards.^15,16^ The study protocol was registered in the OSF Registries on August 4, 2023 (Registration doi: https://doi.org/10.17605/OSF.IO/7SB2F).

### Data Sources and Search Strategy

A comprehensive literature search was conducted without restrictions with EMBASE (Elsevier, Amsterdam, the Netherlands), Ovid MEDLINE (National Institutes of Health, Bethesda, MD), Web of Science Core Collection (Clarivate, Philadelphia, PA), and Cochrane Central (Wiley, Hoboken, NJ), covering all records from their inception to August 20, 2023. Additionally, the first 200 results from Google Scholar were included. The only search filters applied were those excluding conference abstracts and books. To further identify relevant studies, the reference lists of the final included studies were manually reviewed. The search strategy was developed by an expert librarian (Supplemental Table 1, located online at https://doi.org/10.1093/asj/sjae228).

### Study Selection

All retrieved titles and abstracts were independently screened in duplicate by researchers (M.A., G.A., and A.L.) following the eligibility criteria. All provided full texts were similarly reviewed in duplicate. Disagreements were discussed with the fourth researcher (T.M.). All original studies that described investigations of the impact of MFU-V on outcomes related to aesthetics, skin aging, skin quality, satisfaction, and safety were considered for inclusion. The study participants included adults (≥18 years) with a sample size exceeding 10, and there were no restrictions based on participant characteristics, health status, or treated regions. We excluded case reports, reviews, letters to editors, conference abstracts, and studies reporting on the combined effects of MFU-V with other treatments and studies conducted in animals, children, or adolescents.

### Data Extraction and Quality Assessment

Data from the included studies were extracted based on a predesigned Excel form. The primary extracted information included the first author's name, study design, publication year, location, number of participants, sex distribution of the population, participant health status at study entry, age, follow-up duration, ethnicity, skin type, device name and brand, treated area, transducer information, treated depth, outcomes assessment methods, adjustments, and any measure of frequency or association. The quality of included studies was evaluated based on the design of the study with Cochrane Collaboration's tool Risk of Bias 2 (RoB 2) or the risk of bias in nonrandomized studies of interventions (ROBINS-I) tool.^17,18^ Further information is provided in the Appendix, located online at https://doi.org/10.1093/asj/sjae228.

### Data Synthesis

The various reported outcomes, including improvement scales, skin quality parameters, satisfaction, and safety profiles following MFU-V treatment; the number of studies; homogeneity in study designs; assessment tools; and outcome estimates, enabled us to conduct a meta-analysis for an overall/global aesthetic improvement score (as assessed by patients and investigators), patient satisfaction, and pain following MFU-V treatment. Summary measures (eg, proportion and mean values) were combined with the generalized linear mixed model with logit-transformed proportions. The Hartung-Knapp method was utilized to estimate the 95% confidence intervals for random-effects models. Random-effects models account for between-study heterogeneity. Fixed-effects models were presented as sensitivity analyses.

To ensure the homogeneity of studies regarding the assessment tools that quantified overall aesthetic improvement, we specifically included studies that reported on assessment of global/overall aesthetic improvement with either the Global Aesthetic Improvement Scale (commonly categorized as a 5-point scale ranged from worse to very much improved) or a 5-point scale that offered “no change” and “worse” as possible response options for both patients and investigators, and reported the findings as frequency or proportion. For this outcome, we aggregated the results for any improvement category, including mildly improved/improved, moderately/much/markedly improved, and very much improved. Similarly, for the meta-analysis of patient satisfaction, we aggregated the findings for any level of satisfaction, encompassing both very satisfied and satisfied responses (as options provided among the included studies) following MFU-V treatment. Given that some studies included “neutral” as a response option, we took this into account in a following subgroup analysis of this outcome. For pain scores (as a continuous outcome) that were presented as medians, ranges, or 95% confidence intervals, we computed means and standard deviations following the method described previously.^19^ For studies that presented findings separately for each treated site or after applying each transducer without offering an overall pain estimate, we initially aggregated the findings within each of these studies with a fixed-effect model. This aggregated estimate was then utilized for the meta-analysis across all studies.

For randomized clinical trials, we solely extracted the treatment outcomes observed in the MFU-V arm, and so all our meta-analyses included MFU-V pre/post intervention data (Appendix, located online at https://doi.org/10.1093/asj/sjae228). Further analysis details, including subgroup analyses, publication bias assessment, heterogeneity examination, and sensitivity analysis, can be found in the Appendix. All analyses were performed with R version 4.1.3, with the meta and dmetar packages.

## RESULTS

### Eligible Studies

**Figure 1.**
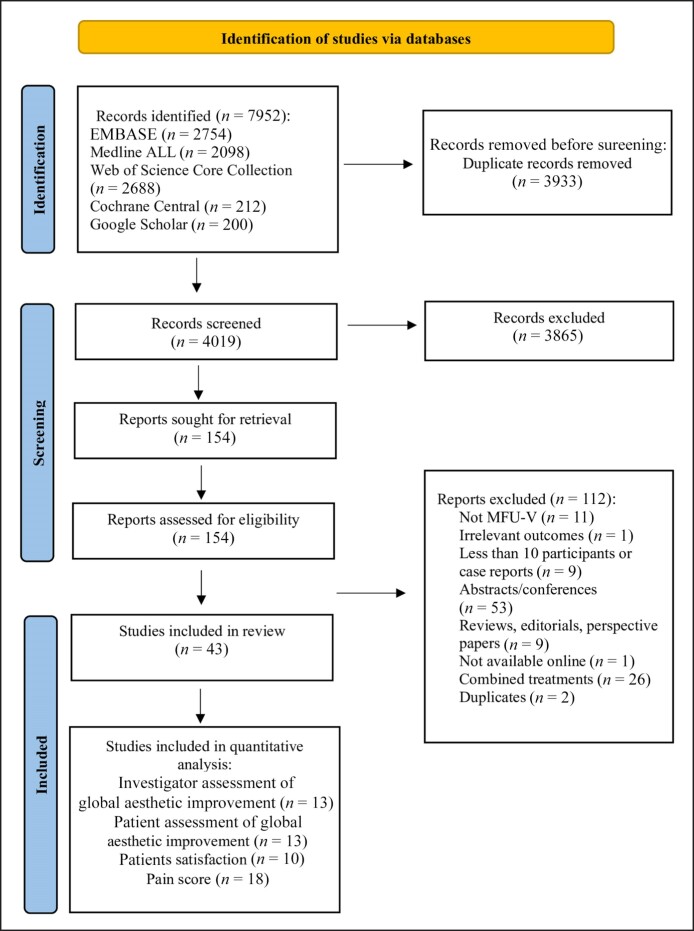
Flowchart of identification, screening, eligibility, inclusion, and exclusion of retrieved studies.

Among 4019 identified references, 42 studies were included. Of these, 23 reported on global/overall aesthetic improvement as scored by investigators and 21 as scored by patients, 18 reported on patient satisfaction, and 33 reported on pain scores following MFU-V treatment. Eleven studies reported on wrinkles, 18 reported on skin firmness and elasticity, and 10 described other skin quality parameters like roughness, texture, and pigmentation. Additionally, 4 studies reported on on eyebrow height in facial regions. For the quantitative analysis, 13 studies were part of the meta-analysis of global/overall aesthetic improvement scored by investigators and 13 scored by patients, 10 for patient satisfaction scores, and 18 for pain scores. The remaining findings contributed to the narrative synthesis (Figure 1).

### Study Characteristics

The included studies were published between 2012 and 2023. Of 42 studies, 2 were controlled clinical trials, 9 were observational studies incorporating both prospective and retrospective cohorts, and the remaining 31 were pre/post designs.^5,10,20-28^ Follow-up durations varied from immediate posttreatment assessments (emphasizing pain or adverse events) to a maximum of approximately 365 days, with around 80% of the studies featuring follow-up periods within 6 months (∼180 days). Sample sizes ranged from 10 to 459 participants. Both males and females were included in 26 studies, and 16 were exclusively focused on females.^5-50^ Studies of facial areas alone included 29% of all studies, 30% considered both neck and facial regions, and 7% reported exclusively on the neck.^5,7-13,20-24,29-40,42-44^ The remaining studies reported on the MFU-V treatment of various other body parts (Table 1, and Supplemental Table 2 located online at https://doi.org/10.1093/asj/sjae228). Additionally, the studies reported on a variety of pain management approaches. Among these, oral medications such as ibuprofen and paracetamol and application of topical creams were the most reported approaches (Supplemental Table 3, located online at https://doi.org/10.1093/asj/sjae228).

**Table 1.** Characteristics of Included Studies by Treated Region

Region	Study characteristics
Facial region^11,12,20,21,29-35^	11 studies | 81.8% pre/post interventional, 18.2% retrospective observational | *n* = 617 | ≥ 74% female | median follow-up: 166 days | 63.66% Ulthera System (Ulthera, Inc., Raleigh, NC), 27.25% Ultraformer III (Classys Inc., Seoul, Korea), 9.09% Microson (Cosmoplus Co, Sungnam, Korea).
Facial and neck regions^7,9,10,22-24,36-39,42-44^	13 studies | 69.3% pre/post interventional, 15.4% prospective observational, 7.65% retrospective observational, 7.65% controlled trial| *n* = 617 | ∼ 90% female | median follow-up: 180 days | 92.3% Ulthera System, 7.7% device not mentioned.
Neck region^5,13,40^	3 studies | 66.6% pre/post interventional, 33.3% controlled trial| *n* = 102 | 93% female | median follow-up: 180 days | Ulthera System.
Décolletage^6,47,48^	3 studies | 100% pre/post interventional| *n* = 155 | 100% female | median follow-up: 180 days | Ulthera System.
Arm^41,51^	2 studies | 100% pre/post interventional| *n* = 96 | 94% female | median follow-up: 183 days | Ulthera System.
Elbow^46^	1 study | 100% pre/post interventional| *n* = 18 | 100% female | median follow-up: 180 days | device not mentioned.
Lower abdomen^49,50^	2 studies | 100% pre/post interventional| *n* = 78 | 100% female | median follow-up: 183 days | 50% Ulthera System, 50% device not mentioned.
Buttocks^14^	1 study | 100% pre/post interventional| *n* = 27 | 96.7% female | median follow-up: 180 days | Ulthera System.
Above knee^8^	1 study | 100% pre/post interventional| *n* = 28 | 96.7% female | median follow-up: 180 days | Ulthera System.
Combination of several regions^25-28,45^	5 studies | 60% retrospective observational, 20% pre/post interventional, 20% prospective observational | *n* = 896 | 96% female | median follow-up: 152 days | 80% Ulthera System, 20% device not mentioned.

### Quality Assessment

**Figure 2.**
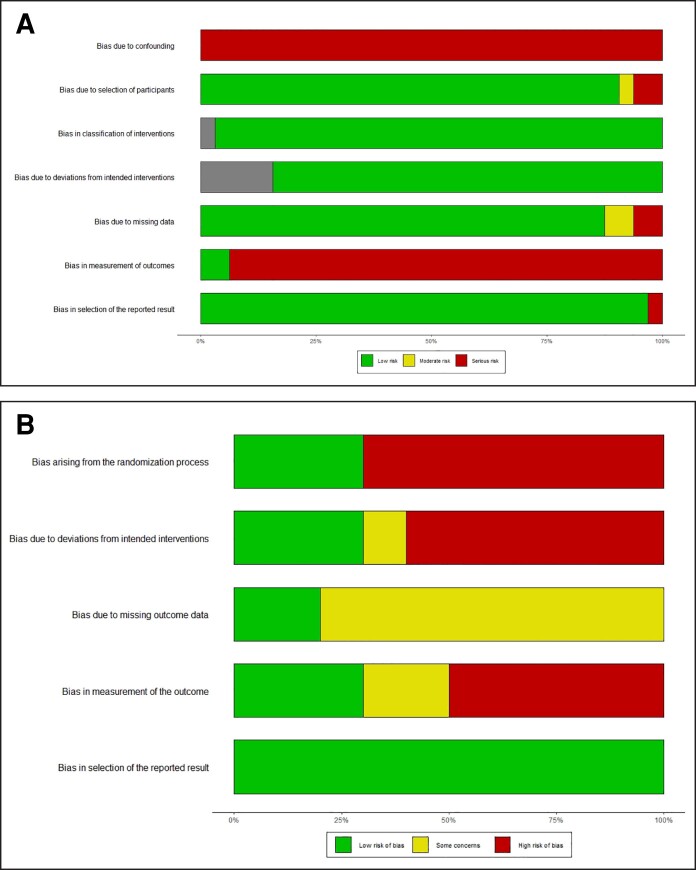
Risk of bias summary of (A) included nonrandomized studies using risk of bias in nonrandomized studies (ROBINS-I) and (B) randomized controlled studies using Cochrane Collaboration's Tool Risk of Bias 2 (ROB2). The shaded regions in (A) indicate “no information” for assessing the corresponding domain in certain studies.

In nonrandomized studies, which include nonrandomized clinical trials, pre/post interventional studies, and observational research, all were subject to confounding bias, and the majority exhibited bias in outcome measurement. Among randomized clinical trials, most concerns arose from the randomization process, deviation in intended intervention, and bias in outcome measurement (Figure 2A, 2B, and Supplemental Tables 4, 5 located online at https://doi.org/10.1093/asj/sjae228).

### Findings of Meta-Analysis

#### Investigator and Patient Overall Aesthetic Improvement Assessment

**Figure 3.**
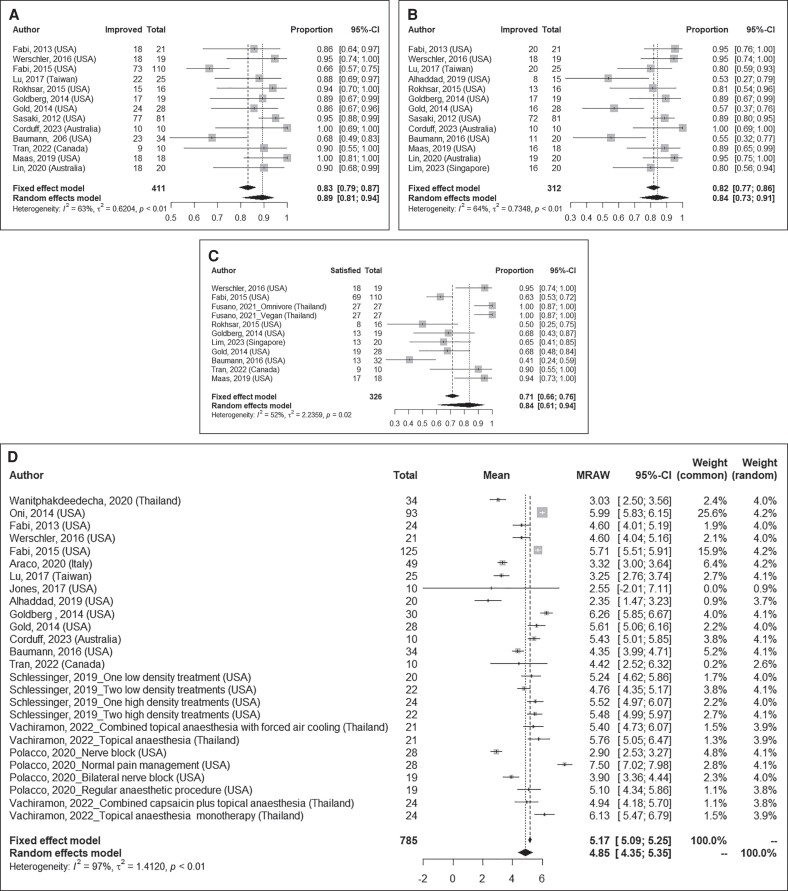
Summary proportions and pooled estimates of (A) investigator global aesthetic improvement, (B) patient global aesthetic improvement, (C) patient satisfaction, and (D) pain following MFU-V treatment. MFU-V, microfocused ultrasound with visualization.

**Figure 4.**
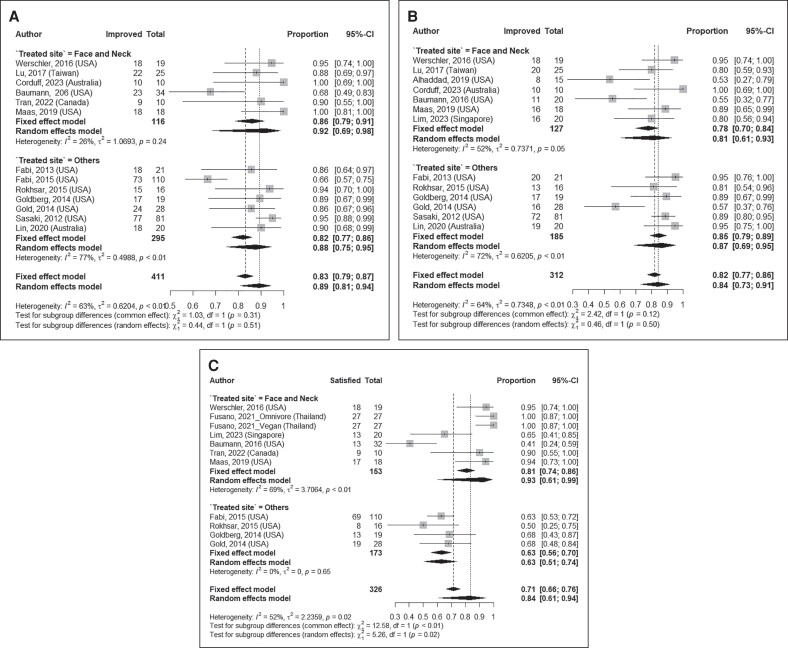

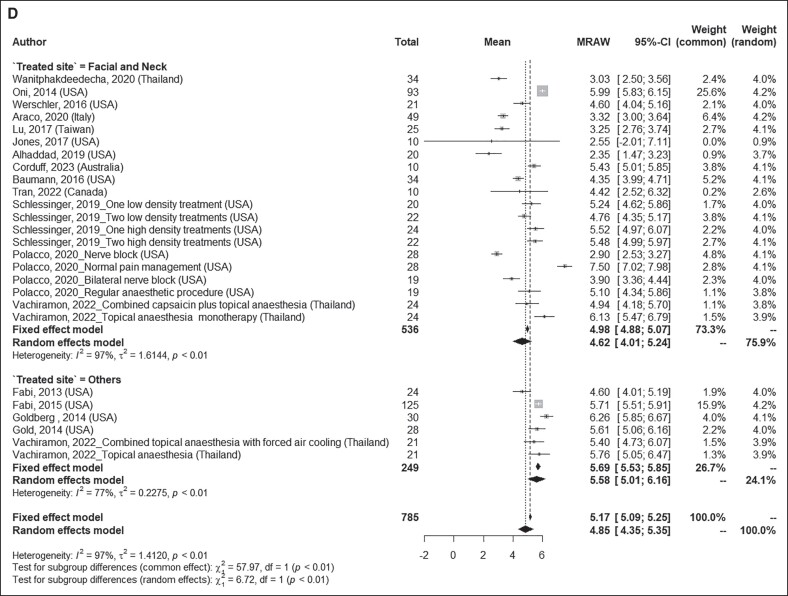
Summary proportions and pooled estimates of (A) investigator global aesthetic improvement, (B) patient global aesthetic improvement, (C) patient satisfaction, and (D) pain following MFU-V treatment per treated site. MFU-V, microfocused ultrasound with visualization.

Pooled results of overall aesthetic improvement in 411 patients, as assessed by investigators, showed that 89% (95% CI: 81%-94%; I^2^: 63%, Figure 3A) of patients were determined to have experienced some level of improvement following MFU-V treatment. Pooled estimates of self-reported overall aesthetic improvement by 312 patients indicated that 84% (95% CI: 73%-91%; I^2^: 64%, Figure 3B) experienced some degree of improvement following treatment. Similar findings were obtained upon analysis by treated areas, including the facial and neck regions and other treated sites (Figure 4A, B).

#### Patient Satisfaction

The meta-analysis, including 326 patients, showed that 84% (95% CI: 61-94%; I^2^: 52%, Figure 3C) expressed satisfaction with the results. The satisfaction was more prominent for patients receiving treatment in the facial and neck regions (pooled estimate: 93% (95% CI: 61%-99%); I^2^: 69%, *n* = 153, Figure 4C) than in other regions (pooled estimate: 63% (95% CI: 51%-74%); I^2^: 0.0%, *n* = 173, Figure 4C).

#### Pain Score

Our meta-analysis, including 785 patients, over a range 0-10 (0 no pain, 10 severe pain), showed a mean pain score of 4.85 (95% CI: 4.35, 5.35; I^2^: 97%, Figure 3D) following MFU-V treatment. Stratifying by treated sites, patients treated in the facial and neck regions reported a mean pain score of 4.62 (95% CI: 4.01, 5.24; I^2^: 97%, *n* = 536, Figure 4D), whereas those treated in other regions reported a mean score of 5.58 (95% CI: 5.01, 6.16; I^2^: 77%, *n* = 249, Figure 4D).

The results of studies that did not meet the eligibility criteria for inclusion in the meta-analysis are outlined in Supplemental Tables 6 and 7 (located online at https://doi.org/10.1093/asj/sjae228); in general, their findings were in line with meta-analysis results.

#### Additional Analyses

The analysis by study location did not show any significant differences by country, except for the meta-analysis on patient satisfaction, which showed more satisfaction for studies conducted in countries other than the USA (Supplemental Figures 1, 2, located online at https://doi.org/10.1093/asj/sjae228). Similarly, the patient satisfaction analysis showed that among studies in which a neutral response was included, 62% (95% CI: 37%-82%; I^2^: 3%, *n* = 172) of participants were satisfied with the results; in contrast to 91% (95% CI: 46%-99%; I^2^: 24%, *n* = 136) in studies that did not include a neutral response (Supplemental Figure 1D).

Meta-regression findings demonstrated that follow-up time did not significantly impact the meta-analyses results (*P* value >.05, Supplemental Table 8, located online at https://doi.org/10.1093/asj/sjae228). As indicated by the Egger test, publication bias was noted for the meta-analysis of investigator global aesthetic improvement assessment and patient satisfaction. The leave-1-out analysis did not show a major influence of any studies in the meta-analyses (Supplemental Figures 3, 4, located online at https://doi.org/10.1093/asj/sjae228).

### Findings of Systematic Review

Given the diverse study designs, varied assessment methods, and differing measures of association within the included studies, conducting a meta-analysis for skin quality and other extracted outcomes was not feasible; therefore, the narrative summarizes the findings.

#### Skin Quality

Wrinkle presence was assessed in 11 studies in various body regions, including 6 studies of the face and/or neck and the remaining of other body parts, at various time points.^6,7,9,11,13,14,22,38,45,47,48^ Several studies reported some degree of improvement after treatment.^6,7,9,11,13,14,38,47,48^ Baseline values were not consistently evaluated or compared in all studies.

A total of 18 studies reported findings on laxity, sagging, firmness, elasticity, skin tightening, and ptosis following MFU-V in various body regions, with the majority, 11 studies, reporting on the face and/or neck, and the remaining on other regions.^5-10,12-14,22-24,27,42,43,46,49,51^ Regardless of the treatment site, studies consistently reported some degree of improvement; however, different measurement methods were applied.

Skin roughness, evenness, texture, smoothness, pigmentation, and pore size, were evaluated in total by 7 studies of the face and/or neck, and 3 of other body regions.^7-9,11,13,14,22,26,34,38^ Among studies, a higher smoothness and evenness were reported at 90 or 180 days after treatment; however, no statistical comparison with baseline values was provided.^7,8,14,22^ Also, 3 studies reported some degree of improvement in the mean value of skin roughness, pigmentation, and texture, and another study on skin texture did not describe any change.^9,11,13,26,38^ Among these studies, only 2 reported a comparison with baseline values that showed skin roughness significantly reduced 6 months after treatment.^11^ Findings of 3 studies on pore size reported improvement in 3 to 6 months after treatment; however, only 1 reported within-group comparisons for which the change was insignificant (Supplemental Table 9, located online at https://doi.org/10.1093/asj/sjae228).^9,34,38^

#### Adverse Events

Erythema, edema, and swelling followed by bruising and tenderness were among the most reported adverse events, all mild or moderate in severity. All studies reported no acute skin damage or long-term sequelae such as nerve or muscle dysfunction, scarring, ulceration, hypopigmentation, or hyperpigmentation (Supplemental Table 10, located online at https://doi.org/10.1093/asj/sjae228).

#### Other Outcomes

Four studies reported findings on eyebrow height.^9,11,38,45^ Other outcomes, such as facial volume, lightening, area and severity of melasma index, bulging severity score, appearance of scars/severity of acne scars, submental lift, and arm circumferences were reported by a limited number of studies (Supplemental Tables 11, 12, located online at https://doi.org/10.1093/asj/sjae228).^9,11,29,30,51^

## DISCUSSION

Our findings indicate an aesthetic improvement of over 80%, as assessed by investigators (92% in face and neck and 88% in other treated sites) and patients (81% in face and neck and 87% in other treated sites). Correspondingly, 84% of patients expressed satisfaction with the results overall (93% in face and neck, 63% in other treated sites, 62% when “neutral” was a response option). The study also revealed a mean pain score of 4.87 (4.62 in the face and neck and 5.58 in other treated sites), categorized as moderate. These results aligned with the safety profile, which did not show any serious adverse events.

The mechanisms underlying MFU-V are still undergoing exploration. MFU-V works by raising tissue temperatures, creating small thermal coagulation zones at focal depths of up to 5 mm while maintaining the integrity of the overlying epidermis and papillary dermis.^52,53^ This process is believed to trigger collagen denaturation, contraction, and subsequent neocollagenesis.^52^ Although 2 studies, which did not meet our inclusion criteria, reported an increase in collagen and elastin production with MFU-V, these studies were limited by small sample sizes and lacked a control group.^41,54^ This meta-analysis consistently showed high satisfaction rates, exceeding 80% in facial and neck areas but dropping to 63% in other body parts. When performing subgroup analysis by including neutral responses, the satisfaction rate is closer to 60%. This suggests that when a neutral option is not provided, some patients in the neutral group might be misclassified as satisfied. This misclassification is likely because any degree of satisfaction was considered in such cases. Several factors may contribute to the observed findings on satisfaction. Satisfaction is highly subjective, influenced by personal preferences, perceptions, and previous experiences. Patient goals, previous treatment experiences, and the complexity of treating specific body areas like the knee may impact satisfaction levels.^55^ This observation holds significance, especially considering that “other body parts” encompassed the décolletage, elbow, buttocks, and knee.

Our study also indicates that MFU-V is generally safe, with mild or moderate adverse events. However, it should be noted that in studies not meeting the inclusion criteria and with the technique not explicitly called MFU-V, 2 cases of hyperpigmentation were reported following transcutaneous focused ultrasound treatment with a 7.0 MHz/4.5 mm transducer.^56^ Our analysis also showed a moderate mean pain score following treatment. Factors beyond the technique itself influence posttreatment pain and discomfort. Patient familiarity with cosmetic procedures can affect their adaptation to discomfort. Those with previous experience may handle discomfort better.^57^ Medication use, tailored by clinicians based on patient pain tolerances, also significantly impacts perceived pain after treatment.^48^

The observed heterogeneity in our meta-analyses may stem from several factors. Ethnicity and skin type can influence skin characteristics. Participant characteristics also play a role. Other contributors include variations in sample sizes, follow-up durations, and outcome definitions across studies. Regarding the pain analysis, the included studies employed a variety of pain management approaches. Although subgroup analysis showed consistent results, our findings were limited by study-level data rather than individual patient data. Further research is needed to understand factors that may influence the outcomes of MFU-V treatment.

The current study exhibits several notable strengths. A comprehensive and broad search strategy and screening procedure in duplicate were employed. Furthermore, in our study we went beyond evaluating the impact of MFU-V solely on facial regions; we comprehensively explored its application to other body parts, including the neck, décolletage, abdomen, and arm. We undertook both meta-analysis and subgroup analysis, allowing for the exploration of the varying impact of MFU-V based on the treated area and other factors that could influence the results.

Nevertheless, there also were some limitations. Many studies lacked control groups, hindering robust comparisons and conclusions about MFU-V effectiveness. Additionally, the absence of randomization and blinding procedures in numerous studies raised concerns about measurement biases and confounding factors. Insufficient reporting of participant ethnicity limited our assessment of demographic influences on outcomes. Due to variations in assessment tools and reported estimates, we were unable to include all studies in the analysis of overall aesthetic improvement scores, satisfaction, and pain. However, the findings from these studies were summarized and found to be consistent with the results of the meta-analysis. Furthermore, the heterogeneity in pain management approaches, particularly in medication type, application methods, and dosages, prevented us from conducting a subgroup analysis based on anesthetic usage. Additionally, few studies accounted for confounders, with only a subset conducting multivariable regression or providing correlations between outcomes and relevant variables. The severity of skin laxity of the patients at the beginning of a study may influence the effectiveness of the therapy.^31,58^ It can also reduce the perceived improvement following MFU-V. Additionally, body mass index (BMI) can impact the therapeutic effect of cosmetic procedures. However, among the included studies, only 2 studies reported on the influence of BMI.^22,31^ Such factors should be assessed at study entry and monitored throughout the research period, and their potential impact on study outcomes should be explored.

The interchangeable names of ultrasound approaches in clinical settings can cause confusion. Careful consideration is needed in future studies to specify the focused ultrasound technique clearly. The challenges faced in defining study design due to lack of clarity pose a risk of misclassification. Improved clarity in describing statistical methodologies and reported estimations would enhance the interpretability of future research. Additionally, offering patients a full range of response options, including neutral or no change, when assessing outcomes, such as with satisfaction and aesthetic improvement scales, may lead to less misclassification and more accurate and precise results. Furthermore, the predominant research focus has centered on facial and neck regions. Although MFU-V has obtained FDA clearance to lift the neck, chin, and brow and improve lines and wrinkles on the upper chest, its utilization has been noted in areas beyond this. Well-designed randomized controlled clinical trials addressing the limitations identified in the present study are needed to comprehensively grasp the diverse range of potential applications and the effectiveness of MFU-V.

Despite these limitations, this study contributes meaningfully to the existing knowledge base by providing a more holistic evaluation of MFU-V across multiple body areas, not just the face. This broader scope helps fill a gap in the literature and offers insights into how MFU-V may be applied in different clinical contexts. Moreover, our findings enhance our understanding of how specific factors, such as treatment area and assessment methods, may influence the outcomes of MFU-V treatments.

## CONCLUSIONS

Our analysis demonstrated an enhancement in global aesthetic score and patient satisfaction, accompanied by a moderate pain score, following MFU-V treatment. The evidence showed that skin quality parameters aligned with these outcomes and suggested a generally favorable safety profile. However, future studies should address the gaps identified in the current research, including the role of participant characteristics, confounders, and variability in assessment tools. Well-designed, controlled trials are needed to further explore MFU-V's efficacy and safety, with attention to these factors to refine and optimize its clinical applications.

## Supplemental Material

This article contains supplemental material located online at https://doi.org/10.1093/asj/sjae228.

## Supplementary Material

sjae228_Supplementary_Data
