# Supplementary material for: Microfocused Ultrasound With Visualization (MFU-V) Effectiveness and Safety: A Systematic Review and Meta-Analysis
Source: Aesthet Surg J. 2024 Nov 14;45(3):NP86–94. doi: 10.1093/asj/sjae228 (PMC11834976; doi:10.1093/asj/sjae228)
Supplement: sjae228_Supplementary_Data [file sjae228_supplementary_data.docx]

**Appendix: Microfocused ultrasound with visualization (MFU-V) effectiveness and safety: A systematic review and meta-analysis**

**Supplemental Table 1.** Designed search strategies.

| Database | Strategy |
| --- | --- |
| Embase | ('microfocused ultrasound'/exp OR 'microfocused ultrasound with visualization'/exp OR 'ultherapy'/exp OR 'high intensity focused ultrasound'/exp OR (((focused* OR microfocused) NEAR/3 (ultrasound*)) OR ultherap* OR MFU-V OR MFUV OR IFUS OR HIFU):ab,ti,kw) AND ('body regions'/exp OR 'skin'/exp OR 'face'/exp OR 'hand'/exp OR (skin OR derm* OR epiderm* OR dermal* OR wrinkle* OR face OR facial OR hand OR hands OR cheek* OR buttock* OR jaw* OR neck OR elbow* OR foot OR feet OR decolle* OR arm OR arms OR leg OR legs OR forehead* OR ear OR ear OR fold OR folds OR line OR lines OR chin OR eyelid* OR lip OR lips OR abdom* OR nonfacial* OR tigh OR tighs OR chest OR chests OR orbit* OR periorbit* OR submentum OR submental OR brow OR brows):ab,ti,kw) NOT ([Conference Abstract]/lim OR [preprint]/lim) |
| Medline | ("High-Intensity Focused Ultrasound Ablation"/ OR (((focused* OR microfocused) ADJ3 (ultrasound*)) OR ultherap* OR MFU-V OR MFUV OR IFUS OR HIFU).ab,ti,kf.) **AND** (exp "Body Regions"/ OR exp "Skin"/ OR (skin OR dermis OR epiderm* OR derm* OR wrinkle* OR face OR facial OR hand OR hands OR cheek* OR buttock* OR jaw* OR neck OR elbow* OR foot OR feet OR decolle* OR arm OR arms OR leg OR legs OR forehead* OR ear OR ear OR fold OR folds OR line OR lines OR chin OR eyelid* OR lip OR lips OR abdomen* OR abdom* OR nonfacial* OR tigh OR tighs OR chest OR chests OR orbit* OR periorbit* OR submentum OR submental OR brow OR brows).ab,ti,kf.) NOT (news OR congres* OR abstract* OR book* OR chapter* OR dissertation abstract*).pt. |
| Web of Science | TS=(((((focused* OR microfocused) NEAR/2 (ultrasound*)) OR ultherap* OR MFU-V OR MFUV OR IFUS OR HIFU)) AND ((skin OR derm* OR epiderm* OR dermal* OR wrinkle* OR face OR facial OR hand OR hands OR cheek* OR buttock* OR jaw* OR neck OR elbow* OR foot OR feet OR decolle* OR arm OR arms OR leg OR legs OR forehead* OR ear OR ear OR fold OR folds OR line OR lines OR chin OR eyelid* OR lip OR lips OR abdom* OR nonfacial* OR tigh OR tighs OR chest OR chests OR orbit* OR periorbit* OR submentum OR submental OR brow OR brows))) **AND** DT=(Article OR Review OR Letter OR Early Access) |
| Cochrane | ((((focused* OR microfocused) NEAR/3 (ultrasound*)) OR ultherap* OR MFU NEXT/1 V OR MFUV OR IFUS OR HIFU):ab,ti,kw) **AND** ((skin OR derm* OR epiderm* OR dermal* OR wrinkle* OR face OR facial OR hand OR hands OR cheek* OR buttock* OR jaw* OR neck OR elbow* OR foot OR feet OR decolle* OR arm OR arms OR leg OR legs OR forehead* OR ear OR ear OR fold OR folds OR line OR lines OR chin OR eyelid* OR lip OR lips OR abdom* OR nonfacial* OR tigh OR tighs OR chest OR chests OR orbit* OR periorbit* OR submentum OR submental OR brow OR brows):ab,ti,kw) |
| Google Scholar | 'focused\|microfocused ultrasound'\|ultherapy skin\|dermal\|dermis\|wrinkle\|face\|nonfacial\|hand\|cheek\|buttock\|neck\|arm\|tigh\|chest\|decolletage\|abdomen\|eyelid\|lip\|chest\|brow |

**Supplemental Information 1:**

Quality of the controlled clinical trials were assessed using Cochrane Collaboration’s Tool Risk of Bias 2 (RoB 2), evaluating biases arising from the randomization process, intended intervention, missing data, measurements of the outcomes, and selection of the reported results ^1^. The risk of bias judgments for each domain are “low risk of bias,” “some concerns,” or “high risk of bias”.

Risk of bias in non-randomized studies of interventions (ROBINS-I) tool was used to assess the quality of non-randomized studies. ROBINS-I evaluates the quality of studies based on biases that may arise at different study phases including pre-intervention (confounding and selection of participants), at intervention (classification of intervention), and post-intervention (deviation from intended intervention, missing data, measurements of the outcomes, and selection of the reported outcomes) ^2^. In ROBINS-I, the risk of bias judgments for each domain are “no information,” “critical risk of bias,” “serious risk of bias,” “moderate risk of bias,” or “low risk of bias.”

**Supplemental Information 2:**

Out of 42 included studies, some were initially designed as controlled clinical trials which aimed to explore the effectiveness of MFU-V in comparison with MFU-V and different pain management protocols ^3-7^, compare the effectiveness of different density of MFU-V ^8-10^, and compare the impact of this treatment in vegan and omnivore participants ^11^. Given that both arms in these studies received MFU-V, we treated each arm as an independent effect estimate, considering the design of each estimate as a pre-post interventional study.

Additionally, two studies compared the effectiveness of MFU-V with combined application of MFU-V and hyaluronic acid or autologous blood cell factors ^12,13^. Similarly, only the findings from the MFU-V arm were included in the current study, resulting in a higher number of effect estimates than the actual number of studies.

**Supplemental Information 3:**

We conducted subgroup analyses for each outcomes when 10 or more studies were included in the meta-analysis ^14^. Subgroup analyses were performed based on study locations (USA and Others) and treated areas (Face and neck, and Others). For patients’ satisfaction, since different studies used various scales to assess outcomes, with some incorporating a ‘Neutral’ response and others not, we analyzed sub-groups based on whether the studies included scales with the neutral response. Also, a meta-regression analysis was performed with follow-up time duration (day) as the predictor.

Publication bias was assessed when at least 10 studies were available with Egger’s test and by visually exploring funnel plots for asymmetry ^15,16^. Heterogeneity between studies was assessed using the I2 ^17^. To assess the influence of individual studies on the overall results (sensitivity analysis), a leave-one-out analysis was conducted which involves systematically leaving out each study one at a time and re-computing the meta-analysis summary measures.

**Supplemental Table 2.** Characteristics of included studies examining the aesthetic effectiveness, patients’ satisfaction, and safety following treatment with MFU-V.

| **Study characteristics** | | | | **Participants characteristics** | | | | | **Exposure/Intervention** | | | **Outcomes** |
| --- | --- | --- | --- | --- | --- | --- | --- | --- | --- | --- | --- | --- |
| **Author, year** | **Country** | **Design** | **Follow-up** | **No. participants^1^** | **Sex (%F)** | **Health status/ characteristics** | **Age^2^** | **Ethnicity** | **Treated cites** | **Device (Brand)** | **Treatment** | **Categories** |
| ***Facial regions*** | | | |  | | | | | | | | |
| Lim et al., 2023 ^18^ | Singapore | Interventional (Pre-Post) | 5 m | 20 | B (95) | Mixed melasma on both cheeks. | 53.5 | Chinese | Cheeks | Ulthera® System (Ulthera, Inc., Raleigh, NC, USA) | Transducers: 4 MHz, 7 MHz, 10 MHz; Focal depths: 4.5mm, 3mm, 1.5mm; Lines: 90 to 210 lines per treatment site. | Aesthetic; Satisfaction; Safety |
| Maas et al., 2019 ^19^ | USA | Interventional (Pre-Post) | 180 d | 18 | B (60) | Moderate-to-severe atrophic acne scars, required to be predominantly rolling- and boxcar-type scars, affecting an area ≥5.0 cm^2^, with few or no ice-pick scars present. | 44.5±11.5 | Caucasian, Asian, Native American/ Alaska | Cheeks and/or temples | Ulthera® System (Ulthera, Inc., Raleigh, NC, USA) | Transducers: 7 MHz, 10 MHz; Focal depths: 3 mm, 1.5 mm; Lines: 60 total treatment lines. | Aesthetic; Satisfaction; Safety |
| Jeon et al., 2020 ^20^ | South Korea | Observational (Retrospective) |  | 191 | B (84.8) | Lower eyelid fat bulging | 45 | NM | Lower eyelid | Ultraformer III (Classys Inc., Seoul, Korea) | Supine position:  Transducers: Either 7 MHz or 4 MHz; Focal depth: 3.0-mm or 4.5-mm;  Sitting position:  Transducers: 7 MHz, 4 MHz; Focal depth: 3 mm, 4.5 mm  Lines: NM. | Aesthetic;  Safety |
| Wanitphakdeedecha et al., 2020 ^21^ | Thailand | Interventional (Pre-Post) | 6 m | 34 | B (85.3) | With facial laxity | 35.41±6.31 | NM | Upper face | Ultraformer III (Classys Inc., Seoul, Korea) | Transducers: 5.5 MHz; Focal depth: 2 mm; Lines: 140. | Aesthetic;  Safety |
| Oni et l., 2014 ^22^ | USA | Interventional (Pre-Post) | 90 d | 93 | B (84.47) | NM | 49.2 | NM | Lower face | Ulthera® System (Ulthera, Inc., Raleigh, NC, USA) | Transducers: 4 MHz, 7.5 MHz; Focal depth: 4.5 mm, 3 mm; Lines: 295. | Aesthetic;  Safety |
| Lee et al,. 2015 ^23^ | South Korea | Interventional (Pre-Post) | 4 m | 38 | B (97.3) | With sagging and laxity of the facial skin | 46 | NM | Face | Ultraformer III (Classys Inc., Seoul, Korea) | Transducers: 4 MHz, 7.5 MHz; Focal depth: 4.5 mm, 3 mm; Lines: 300. | Aesthetic;  Safety |
| Yalici-Armagan et al., 2020 ^24^ | Turkey | Observational (Retrospective) | Median:  4.3  (3-7.4) | 24 | B (96) | Undergone the therapy for skin tightening | 52.5±7.5 | NM | Lower face | Microson (Cosmoplus Co, Sungnam, Korea) | Transducers: 4 MHz, 7.5 MHz; Focal depth: 4.5 mm, 3 mm; Lines: NM. | Aesthetic;  Safety |
| Araco et al., 2020 ^25^ | Italy | Interventional (Pre-Post) | 180 d | 49 | B (95.9) | Soft-to-moderate skin laxity and ptosis of the face and neck | 52.8±7.43 | Caucasian, Asian | Full face treatment except for the upper one-third | Ulthera® System (Ulthera, Inc., Raleigh, NC, USA) | Transducers: 4 MHz, 7 MHz, 10 MHz; Focal depths: 4.5 mm, 3 mm, 1.5 mm; Lines: 1200 lines (400 for each transducer). | Aesthetic; Satisfaction; Safety |
| Yusova et al., 2019 ^12^ | Russia | Interventional (Pre-Post) | 6 m | 16 | B (94.3) | Individuals with involutional skin changes | 40 - 50 | NM | Lower third of the face, submandibular space | NM | Transducers: 7 MHz; Focal depths: 3 mm; Lines: 310. | Aesthetic |
| Vachiramon et al., 2021 ^13^ | Thailand | Interventional (Pre-Post) | 4 m | 46 | B (69.6) | With enlarged skin pores evenly on both cheeks | 36.7±8.61 | NM | Cheeck | Ulthera® System (Ulthera, Inc., Raleigh, NC, USA) | Transducers: 10 MHz; Focal depths: 1.5 mm; Lines: 50. | Aesthetic; Satisfaction; Safety |
| Schlessinger et al., 2019 ^8^ | USA | Interventional (Pre-Post) _  ***One low-density treatment*** | 365 d | 20 | B (NM) | Healthy adults with a clinical diagnosis of erythematotelangiectatic rosacea | 49.2±9.4 | White | Cheeks, chin, nose, central forehead | Ulthera® System (Ulthera, Inc., Raleigh, NC, USA) | Transducers: 7MHz, 4 MHz, 10MHz; Focal depths: 3 mm, 4.5 mm, 1.5 mm; Lines: minimum of 15 treatment lines per treatment square. | Aesthetic; Safety |
|  |  | Interventional (Pre-Post)_  ***Two low-density treatments*** | 365 d | 22 | B (NM) | Healthy adults with a clinical diagnosis of erythematotelangiectatic rosacea | 51.5±9 | White | Cheeks, chin, nose, central forehead | Ulthera® System (Ulthera, Inc., Raleigh, NC, USA) | Transducers: 7MHz, 4 MHz, 10MHz; Focal depths: 3 mm, 4.5 mm, 1.5 mm; Lines: minimum of 30 treatment lines per treatment square. | Aesthetic; Safety |
|  |  | Interventional (Pre-Post)_  O***ne high-density treatments*** | 365 d | 24 | B (NM) | Healthy adults with a clinical diagnosis of erythematotelangiectatic rosacea | 48.9±11.4 | White | Cheeks, chin, nose, central forehead | Ulthera® System (Ulthera, Inc., Raleigh, NC, USA) | Transducers: 7MHz, 4 MHz, 10MHz; Focal depths: 3 mm, 4.5 mm, 1.5 mm; Lines: minimum of 30 treatment lines per square. | Aesthetic; Safety |
|  |  | Interventional (Pre-Post)_  ***Two high-density treatments*** | 365 d | 22 | B (NM) | Healthy adults with a clinical diagnosis of erythematotelangiectatic rosacea | 49.6±8.4 | White | Cheeks, chin, nose, central forehead | Ulthera® System (Ulthera, Inc., Raleigh, NC, USA) | Transducers: 7MHz, 4 MHz, 10MHz; Focal depths: 3 mm, 4.5 mm, 1.5 mm; Lines: minimum of 60 treatment lines per square. | Aesthetic; Safety |
| ***Facial and Neck regions*** | | | |  | | | | | | | | |
| Fabi et al., 2014 ^26^ | USA | Observational (Retrospective) | 180 d | 45 | F (100) | Healthy women | 58 | Caucasian, Hispanic,  Native Hawaiian  or Pacific, Islander, Asian, Mixed race. | Face and upper neck | Ulthera® System (Ulthera, Inc., Raleigh, NC, USA) | Transducers: 4 MHz, 7 MHz; Focal depths: 4.5mm, 3mm; Lines: 370–420 treatment lines. | Aesthetic;  Safety |
| Shome et al., 2019 ^27^ | India | Interventional (Pre-Post) | 1 y | 50 | B (52) | Mild sagging of skin in the lower half of the face and neck region | F: 38.4±1.2; M: 36.76±2.4 | Indian | Lower half of the face and neck region | Ulthera® System (Ulthera, Inc., Raleigh, NC, USA) | Transducers: 4 MHz, 7 MHz, 10 MHz; Focal depths: 4.5mm, 3mm, 1.5mm; Lines: 500 exposure lines (range: 480–700). | Aesthetic;  Safety |
| Corduff et al., 2023 ^28^ | Australia | Interventional (Pre-Post) | 6-10 m | 10 | B (90) | Mild-to-moderate skin and fibromuscular laxity and neck fat thickness. | 31 - 61 | NM | Full-face and upper neck | NM | Transducers: 4.4 MHz, 7 MHz; Focal depths: 4.5mm, 3 mm; Line: average 280 lines. | Aesthetic;  Safety |
| Harris et al., 2015 ^29^ | USA | Interventional (Pre-Post) | 180 d | 52 | B (98) | Adults with Fitzpatrick skin types III to VI. | 53 | African American/black, Asian,  Hispanic/Latino, Other | Facial and neck areas | Ulthera® System (Ulthera, Inc., Raleigh, NC, USA) | Transducers: NM; Focal depths: NM; Line: approximately 370 lines. | Safety |
| Kerscher et al., 2019 ^30^ | Germany | Observational (Prospective) | 12 w | 22 | F (100) | Moderate-to-severe skin laxity in the submental region and a body mass index of 20–30 kg/m² | 52.32± 9.31 | NM | Submental region and contour the jawline | Ulthera® System (Ulthera, Inc., Raleigh, NC, USA) | Transducers: 4 MHz, 7 MHz; Focal depths: 4.5 mm, 3 mm; Line: 350 and 270 lines. | Aesthetic;  Safety |
| Kerscher et al., 2019 ^31^ | Germany | Observational (Prospective) | 24 w | 22 | F (100) | Moderate-to-severe skin laxity | 52.32± 9.32 | NM | Lower face and submental  region | Ulthera® System (Ulthera, Inc., Raleigh, NC, USA) | Transducers: 4 MHz, 7 MHz; Focal depths: 4.5 mm, 3 mm; Line: 350 and 270 lines. | Aesthetic;  Safety |
| Werschler et al., 2016 ^32^ | USA | Interventional (Pre-Post) | 1 y | 20 | B (85) | Healthy adults with skin laxity on their face and neck | 47 | Caucasian, Hispanic/Latino | Lower face and neck | Ulthera® System (Ulthera, Inc., Raleigh, NC, USA) | Transducers: 4 MHz, 7 MHz, 10 MHz; Focal depths: 4.5 mm, 3 mm, 1.5 mm; Line: NM. | Aesthetic; Satisfaction; Safety |
| Lu et al., 2017 ^33^ | Taiwan | Interventional (Pre-Post) | 180 d | 25 | B (92) | With facial laxity | 53.3±6 | Asian | Face and neck | Ulthera® System (Ulthera, Inc., Raleigh, NC, USA) | Transducers: 4 MHz, 7.5 MHz; Focal depths: 4.5 mm, 3 mm; Line: 800. | Aesthetic;  Safety |
| Alhaddad et al., 2019 ^34^ | USA | Interventional (Controlled_Split face) | 180 d | 20 (20/20) | F (100) | With symmetrical mild to moderate skin laxity of the face and upper neck | 52.6±7.3 | NM | Face and upper neck | Ulthera® System (Ulthera, Inc., Raleigh, NC, USA) | **MFU-V:**  Transducers: 4 MHz, 7 MHz, 10 MHz; Focal depths: 4.5 mm, 3 mm, 1.5 mm; Line: 195 (deeper tissue), 205 (superficial tissu).  **MRF:**  600 pulses, level 2-4, average Level 3 | Aesthetic;  Safety |
| Fusano et al., 2021 ^11^ | Italy | Interventional (Pre-Post)_ ***Vegan*** | 6 m | 27 | F (100) | With skin laxity of lower face and neck | 50.4±3.85 |  | Lower face (cheeks, submental area, mandibular lines), neck | Ulthera® System (Ulthera, Inc., Raleigh, NC, USA) | Transducers: 4 MHz, 7 MHz, 10 MHz; Focal depths: 4.5 mm, 3 mm, 1.5 mm; Line: NM. | Aesthetic; Satisfaction; Safety |
|  |  | Interventional (Pre-Post)_ ***Omnivore*** | 6 m | 27 | F (100) | With skin laxity of lower face and neck | 50.55±3.67 | NM | Lower face (cheeks, submental area, mandibular lines), neck | Ulthera® System (Ulthera, Inc., Raleigh, NC, USA) | Transducers: 4 MHz, 7 MHz, 10 MHz; Focal depths: 4.5 mm, 3 mm, 1.5 mm; Line: NM. | Aesthetic; Satisfaction; Safety |
| Tran et al., 2022 ^3^ | Canada | Interventional (Pre-Post)_  ***MFU-V*** | 3 m | 10 | F (100) | With mild-to-moderate lower face and neck laxity | 38-59 | NM | Lower face, neck | Ulthera® System (Ulthera, Inc., Raleigh, NC, USA) | Transducers: NM; Focal depths: 4.5 mm, 3 mm; Line: NM. | Aesthetic; Satisfaction; Safety |
|  |  | Interventional (Pre-Post)_  ***Nitrous Oxide System During MFU-V*** | 3 m | 10 | F (100) | With mild-to-moderate lower face and neck laxity | 38-59 | NM | Lower face, neck | Ulthera® System (Ulthera, Inc., Raleigh, NC, USA) | Transducers: NM; Focal depths: 4.5 mm, 3 mm; Line: NM. | Aesthetic; Satisfaction; Safety |
| Polacco et al., 2020 ^5^ | USA | Interventional (Pre-Post)_  ***Nerve block*** | 6 d | 28 | B (89.3) | NM | 54 | NM | Face and neck | Ulthera® System (Ulthera, Inc., Raleigh, NC, USA) | Transducers: Face:4-MHz, Neck:7 MHz; Focal depth: Face: 4.5 mm, Neck: 3.0 mm; Line: NM | Safety |
|  |  | Interventional (Pre-Post)_  ***Normal pain management*** | 6 d | 28 | B (89.3) | NM | 54 | NM | Face and neck | Ulthera® System (Ulthera, Inc., Raleigh, NC, USA) | Transducers: Face:4-MHz, Neck:7 MHz; Focal depth: Face: 4.5 mm, Neck: 3.0 mm; Line: NM | Safety |
|  |  | Interventional (Pre-Post)_  ***Bilateral nerve block*** | 6 d | 19 | B (83.7) | NM | 53 | NM | Face and neck | Ulthera® System (Ulthera, Inc., Raleigh, NC, USA) | Transducers: Face:4-MHz, Neck:7 MHz; Focal depth: Face: 4.5 mm, Neck: 3.0 mm; Line: NM | Safety |
|  |  | Interventional (Pre-Post)_  ***Regular anaesthetic procedure*** | 6 d | 18 | B (83.7) | NM | 54 | NM | Face and neck | Ulthera® System (Ulthera, Inc., Raleigh, NC, USA) | Transducers: Face:4-MHz, Neck:7 MHz; Focal depth: Face: 4.5 mm, Neck: 3.0 mm; Line: NM | Safety |
| Palm et al., 2018 ^7^ | USA | Interventional (Pre-Post)_  ***1 application of 4% lidocaine/ 1 of 20% benzocaine*** | Immediate assessments | 14 | F (100) | NM | 51.7 | Caucasian, Asian, Native American. | Face, neck | Ulthera® System (Ulthera, Inc., Raleigh, NC, USA) | Transducers: 4 MHz, 7 MHz, 7 MHz; Focal depths: 4.5 mm, 4.5 mm, 3 mm; Lines; 355. | Safety |
|  |  | Interventional (Pre-Post)_  ***2 applications of lidocaine 23%/tetracaine 7%*** | Immediate assessments | 14 | F (100) | NM | 51.7 | Caucasian, Asian, Native American. | Face, neck | Ulthera® System (Ulthera, Inc., Raleigh, NC, USA) | Transducers: 4 MHz, 7 MHz, 7 MHz; Focal depths: 4.5 mm, 4.5 mm, 3 mm; Lines; 355. | Safety |
| ***Neck*** | | | |  | | | | | | | | |
| Jones et al., 2017 ^35^ | USA | Interventional (Controlled) | 180 d | 20 (10/10) | B (95) | Moderate neck skin laxity | 55±6.98 | NM | Neck | Ulthera® System (Ulthera, Inc., Raleigh, NC, USA) | **MFU-V:**  Transducers: 4 MHz, 7 MHz; Focal depths: 4.5 mm, 3 mm; Line: 265.  **MRF:**  A uniform internal temperature of 60 C was achieved while keeping the surface skin temperature below 43C. | Aesthetic; Satisfaction; Safety |
| Baumann et al., 2016 ^36^ | USA | Interventional (Pre-Post)_ ***Group A*** | 180 d | 34 | B (94.11) | With skin laxity in the lower face and neck | 55.1 | Caucasian, Hispanic/Latino. | Submental, Submandibular areas, Lower neck | Ulthera® System (Ulthera, Inc., Raleigh, NC, USA) | Submental and submandibular areas: Transducers: 4 MHz, 7 MHz; Focal depths: 4.5 mm, 3 mm.  The lower neck:  Transducer 7 MHz, Focal depths: 3 mm.  Lines: 246-296 lines. | Aesthetic; Satisfaction; Safety |
| Vachiramon et al., 2020 ^6^ | Thailand | Interventional (Pre-Post)_ ***Topical anaesthesia monotherapy*** | Immediate assessments | 24 | B (91.67) | With neck laxity | 44.86±5.8 | NM | Neck | Ulthera® System (Ulthera, Inc., Raleigh, NC, USA) | Transducers: 4.5MHz, 7 MHz; Focal depths: 4.5 mm, 3 mm; Lines: In total 90. | Safety |
|  |  | Interventional (Pre-Post)_ ***Combined capsaicin plus topical anaesthesia*** | Immediate assessments | 24 | B (91.67) | With neck laxity | 44.86±5.8 | NM | Neck | Ulthera® System (Ulthera, Inc., Raleigh, NC, USA) | Transducers: 4.5MHz, 7 MHz; Focal depths: 4.5 mm, 3 mm; Lines: In total 90. | Safety |
| ***Combination of several body parts*** | | | |  | | | | | | | | |
| Sasaki et al., 2012 ^37^ | USA | Interventional (Pre-Post) | 6 m | 81 | F (100) | Mild-to-moderate crepey skin outside the orbital rims and mild-to-moderate ptosis of skin folds and orbital portions of the underlying fibro-muscular layer orbicularis oculi muscle. | 51.8 (30 - 72) | Caucasians, Hispanics, Asians, African-American | Periorbitum,  Décolletage, Brachium, Hand, Periumbilicus, Buttock, Inner Thigh, Knee | NM | Periorbital area: Transducers: 7 MHz, 10 MHz; Focal depth: 3.0 mm, 1.5 mm; Lines: 15 radial lines across the upper lid-brow complex, 15 cris-crossing lines at the crow’s feet area, and 15 radial lines below the lower lid margin per each transducer.  Décolletage: Transducers: 7 MHz, 10 MHz; Focal depth: 3.0 mm, 1.5 mm  Lines: 30 per transducer.  Brachium: Transducers: 4 MHz, 7 MHz, Focal depth: 4.5 mm, 3 mm  Lines: 30 per transducer.  Periumbilicus: Transducers: 4 MHz, 7 MHz, 10 MHz; Focal depth: 4.5 mm, 3 mm, 1.5 mm; Lines: 30 per transducer.  Inner Thigh: Transducers: 4 MHz, 7 MHz; Focal depth: 4.5 mm, 3 mm; Lines: 30 per transducer.  Knee: Transducers: 4 MHz, 7 MHz; Focal depth: 4.5 mm, 3 mm; Lines: 30 per transducer.  Hand: Transducers: 7 MHz; Focal depth: 3 mm; Lines: 15.  Buttock: Transducers: 4 MHz, 7 MHz; Focal depth: 4.5 mm, 3 mm; Lines: 30 per transducer. | Aesthetic;  Safety |
| Montes et al., 2019 ^38^ | USA | Observational (Retrospective) | 6 m  (At least) | 52 | B (98.1) | NM | 54.6 | NM | Lower face, Submentum, Neck, Eyes, Décolleté | Ulthera® System (Ulthera, Inc., Raleigh, NC, USA) | Transducers: NM; Focal depths: NM; Line: NM. | Aesthetic; Satisfaction; Safety |
| Casabona et al., 2019 ^39^ | Spain | Observational (Retrospective) | 90 d | 20 | F (100) | Recent or old, moderate-to-severe stretch marks on the buttocks, thighs, breasts, or abdomen and a body mass index of < 35kg/m^2^. | 18 - 55 | NM | Breasts, Buttocks, Thighs, Abdomen | Ulthera® System (Ulthera, Inc., Raleigh, NC, USA) | Transducers: 7 MHz, 10 MHz;  Focal depth: 3 Nmm, 1.5 Nmm  Lines: up to 75 parallel treatment lines. | Aesthetic; Satisfaction; Safety |
| Chang et al., 2019 ^40^ | USA | Observational (Retrospective) | 4 m  (At least) | 459 | B (97.5) | NM | 50.2 | Caucasian, Hispanic,  Asian, African,  American, Other/  Multiracial, Unknown | Full face, Full face/neck, Lower face, Lower face/neck, Jawline, Brow, Neck, Chest, Body. | Ulthera® System (Ulthera, Inc., Raleigh, NC, USA) | Transducers: NM;  Focal depth: 3 mm, 1.5 mm  Lines: NM. | Aesthetic; Satisfaction; Safety |
| Sasaki e al., 2017 ^41^ | USA | Observational (Prospective) | 360 d | 284 | B (93) | Healthy | 55.5 | Caucasian, Asian,  Hispanic,  Middle Eastern,  African, American. | Forehead, Periorbitum, Face, Neck, Décolleté | Ulthera® System (Ulthera, Inc., Raleigh, NC, USA) | Forehead and Periorbitum:  Transducers: 4 MHz, 7 MHz, 10 MHz;  Focal depth: 4.5 mm, 3 mm, 1.5 mm:  Lines: 30 lines per transducer.  Midface, neck, and Décolletage:  Transducers: 4 MHz, 7 MHz;  Focal depth: 4.5 mm, 3 mm:  Lines: 30 lines per transducer. | Aesthetic; Safety |
| ***Elbow*** | | | |  | | | | | | | | |
| Rokhsar et al., 2015 ^42^ | USA | Interventional (Pre-Post) | 180 d | 18 | F (100) | Mild-to-moderate elbow skin laxity | 54 | Caucasian, Asian. | Elbow | NM | Transducers: 4.4 MHz, 7 MHz; Focal depths: 4.5mm, 3mm; Lines: 240 lines per elbow. | Aesthetic; Satisfaction; Safety |
| ***Buttocks*** | | | |  | | | | | | | | |
| Goldberg et al., 2014 ^43^ | USA | Interventional (Pre-Post) | 180 d | 27 | B (96.7) | Body mass index ≤ 40 kg/m2 and lax buttock skin. | 46.7 | Caucasian,  African American/Black, Hispanic/Latino. | Buttocks | Ulthera® System (Ulthera, Inc., Raleigh, NC, USA) | Transducers: 4 MHz, 7 MHz; Focal depths: 4.5 mm, 3mm; Lines: NM. | Aesthetic; Satisfaction |
| ***Arm*** | | | |  | | | | | | | | |
| Vachiramon et al., 2021 ^9^ | Thailand | Interventional (Pre-Post)_ ***Single plane*** | 6 m | 27 | B (93.3) | With skin laxity of both upper arms | 43.6±10.4 | NM | Arm | Ulthera® System (Ulthera, Inc., Raleigh, NC, USA) | Transducers: 4 MHz; Focal depths: 4.5 mm; Lines: 200 lines of treatment over two passes. | Aesthetic; Safety |
|  |  | Interventional (Pre-Post)_ ***Dual plane*** | 6 m | 27 | B (93.3) | With skin laxity of both upper arms | 43.6±10.4 | NM | Arm | Ulthera® System (Ulthera, Inc., Raleigh, NC, USA) | Transducers: 4 MHz, 7 MHz; Focal depths: 4.5 mm, 3mm; Lines: 100 lines of treatment per each pass (in total 200 lines). | Aesthetic; Safety |
| Vachiramon et al., 2022 ^4^ | Thailand | Interventional (Pre-Post)***_ Combined topical anaesthesia with forced air cooling*** | Immediate assessments | 21 | B (95.2) | Healthy subjects | 34.67±6.18 | NM | Inner side of arms | Ulthera® System (Ulthera, Inc., Raleigh, NC, USA) | Transducers: 4 MHz, 7 MHz; Focal depths: 4.5 mm, 3 mm; Lines: In total 20. | Safety |
|  |  | Interventional (Pre-Post)***_ Topical anaesthesia*** | Immediate assessments | 21 | B (95.2) | Healthy subjects | 34.67±6.18 | NM | Inner side of arms | Ulthera® System (Ulthera, Inc., Raleigh, NC, USA) | Transducers: 4 MHz, 7 MHz; Focal depths: 4.5 mm, 3 mm; Lines: In total 20. | Safety |
| ***Décolletage*** | | | |  | | | | | | | | |
| Fabi et al., 2020 ^44^ | USA | Interventional (Pre-Post) | 360 d | 15 | F (100) | Seeking improvement of wrinkles in the décolleté area. | 54.8±7.5 | NM | Décolleté area | Ulthera® System (Ulthera, Inc., Raleigh, NC, USA) | Transducers: 4MHz, 7 MHz, and 10 MHz; Focal depth: 4.5 mm, 3.0 mm, 1.5 mm; Lines: NM. | Aesthetic; Satisfaction |
| Fabi et al., 2013 ^45^ | USA | Interventional (Pre-Post) | 180 d | 24 | F (100) | Moderate-to-severe rhytides | 51.3 | NM | Décolletage | Ulthera® System (Ulthera, Inc., Raleigh, NC, USA) | Transducers: 4MHz, 7 MHz; Focal depth: 4.5 mm, 3.0 mm; Lines: 120, 240. | Aesthetic; Safety |
| Fabi et al., 2015 ^46^ | USA | Interventional (Pre-Post) | 180 d | 116 | F (100) | Moderate-to-severe decollate skin lines and wrinkles | 56.7±7.6 | White,  Hispanic/Latino, Other. | Décolletage | Ulthera® System (Ulthera, Inc., Raleigh, NC, USA) | Transducers: 4MHz, 7 MHz, and 10 MHz; Focal depth: 4.5 mm, 3.0 mm, 1.5 mm; Lines: At least 280 lines of MFU-V on 3 treatment planes. | Aesthetic; Satisfaction; Safety |
| ***Abdomen*** | | | |  | | | | | | | | |
| Lin et al., 20202 ^47^ | Australia | Interventional (Pre-Post) | 6 m | 20 | F (100) | Female patients between 6 and 24 months postpartum | 32.7 | Asian, Caucasian. | Lower abdomen | NM | Transducers: 4MHz, 7 MHz, and 10 MHz; Focal depth: 4.5 mm, 3.0 mm, 1.5 mm; Lines: 300. | Aesthetic; Safety |
| Vachiramon et al., 2020 ^10^ | Thailand | Interventional (Pre-Post)_ ***Single plane*** | 6 m | 30 | F (100) | With presence of abdominal laxity | 43.3±8.79 | NM | Lower abdomen | Ulthera® System (Ulthera, Inc., Raleigh, NC, USA) | Transducers: 4 MHz; Focal depth: 4.5 mm; Lines: NM. | Aesthetic; Safety |
|  |  | Interventional (Pre-Post)_ ***Dual plane*** | 6 m | 28 | F (100) | With presence of abdominal laxity | 43.3±8.79 | NM | Lower abdomen | Ulthera® System (Ulthera, Inc., Raleigh, NC, USA) | Transducers: 4 MHz, 7 MHz; Focal depth: 4.5 mm, 3 mm; Lines: 200. | Aesthetic; Safety |
| ***Knee*** | | | |  | | | | | | | | |
| Gold et al., 2014 ^48^ | USA | Interventional (Pre-Post) | 180 d | 28 | F (100) | Mild- to moderate bilateral skin laxity above the knees. | 54 | Caucasian,  African American. | Above knees | Ulthera® System (Ulthera, Inc., Raleigh, NC, USA) | Transducers: 4 MHz, 7 MHz; Focal depths: 4.5 mm, 3 mm; Lines: 480 treatment lines. | Aesthetic; Satisfaction; Safety |

^1^ Number of participants included in the analysis; ^2^ Age reported as either mean, min-max, mean ± SD; MFU-V: micro-focused ultrasound with visualization; MRF: monopolar capacitive-coupled radiofrequency; NM: Not mentioned; B: Both males and females; F: Females.

**Supplemental Table 3.** Summary of anesthetics/pain killer used among the studies.

| Author, year | Anesthetics used |
| --- | --- |
| *Facial regions* | |
| Lim et al., 2023 ^18^ | Topical anesthetic mixture (20% benzocaine, 6% lidocaine and % tetracaine) |
| Maas et al., 2019 ^19^ | Oral ibuprofen (800mg) and a topical anesthetic cream containing 7% lidocaine and 7% tetracaine (Pliaglis® Cream; Galderma Laboratories, LP, Fort Worth, TX) |
| Jeon et al., 2020 ^20^ | The EMLA cream (lidocaine 2.5% and prilocaine 2.5%; Astra Pharmaceutical Products Inc., Westborough, MA, USA) |
| Wanitphakdeedecha et al., 2020 ^21^ | Topical anaesthetic cream EMLA® |
| Oni et l., 2014 ^22^ | Oral medications (5-10 mg of diazepam and 5/325 mg of hydrocodone/acetaminophen [1 or 2 tablets] |
| Lee et al,. 2015 ^23^ | Topical lidocaine |
| Yalici-Armagan et al., 2020 ^24^ | NM |
| Araco et al., 2020 ^25^ | Lormetazepam 2 mg, tramadol 25 mg, and local lidocaine cream |
| Yusova et al., 2019 ^12^ | NM |
| Vachiramon et al., 2021 ^13^ | Local anesthetic cream (EMLA®, Astra Zeneca LP) |
| Schlessinger et al., 2019 ^8^ | Mostly Ibuprofen 800mg |
| *Facial and Neck regions* | |
| Fabi et al., 2014 ^26^ | Topical application of 23% lidocaine/ 7% tetracaine alone, 15% received it in combination with oral diazepam (5–10 mg). The majority of subjects received a combination of topical anesthesia, oral diazepam (5–10 mg), and an intramuscular injection of 50 to 100 mg of meperidine and 50 mg of hydroxyzine |
| Shome et al., 2019 ^27^ | Topical anesthetic ointment (7%, lidocaine–prilo- caine) |
| Corduff et al., 2023 ^28^ | NM |
| Harris et al., 2015 ^29^ | NM |
| Kerscher et al., 2019 ^30^ | NM |
| Kerscher et al., 2019 ^31^ | 800 mg ibuprofen |
| Werschler et al., 2016 ^32^ | Oral tramadol with acetaminophen |
| Lu et al., 2017 ^33^ | Topical anesthesia containing 2.5% lidocaine and 2.5% prilocaine and oral analgesics with ibuprofen 800 mg |
| Alhaddad et al., 2019 ^34^ | 60 mg IM ketorolac and 7% lidocaine–tetracaine topical ointment |
| Fusano et al., 2021 ^11^ | Ibuprofen 400 mg |
| Tran et al., 2022 ^3^ | No pain killer |
| Polacco et al., 2020 ^5^ | Nerve blocks were performed by administering 1% lidocaine with 1:100,000 epinephrine via 31-gauge, 1-mL tuberculin syringes in volumes of 0.25 to 0.5 mL at each site outlined |
| Palm et al., 2018 ^7^ | pretreatment with 50 mg IM meperidine, 25 mg IM promethazine, and 5 mg oral diazepam (for all) + a single application of 4% lidocaine followed by a single application of 20% benzocaine |
| *Neck* |  |
| Jones et al., 2017 ^35^ | 60 mg IM ketorolac tromethamine |
| Baumann et al., 2016 ^36^ | Not specified. [Each subject received a pretreatment pain medication at the discretion of the investigator] |
| Vachiramon et al., 2020 ^6^ | A compounded mixture of 2.5% lidocaine and 2.5% prilocaine |
| *Combination of several body parts* | |
| Sasaki et al., 2012 ^37^ | Beginning with oral non-steroidal anti-inflammatory drugs, pain and sedative medications, distractive hand/foot massages, reducing skin temperatures by an air coolant device, lowering the energy settings either by one level for each transducer, or shortening the length of treatment lines. The usage of topical analgesic gels in a few patients. |
| Montes et al., 2019 ^38^ | NM |
| Casabona et al., 2019 ^39^ | NM |
| Chang et al., 2019 ^40^ | Oral anxiolytic and mild narcotic (Valium, Demerol) |
| Sasaki e al., 2017 ^41^ | use of an oral analgesic (Ibuprofen 800 mg) and a distractive vibrating hand device. |
| *Elbow* | |
| Rokhsar et al., 2015 ^42^ | Oral premedications included ibuprofen 400 mg and/or 800 mg with or without lorazepam 2 mg |
| *Buttocks* | |
| Goldberg et al., 2014 ^43^ | Topical betacaine/lidocaine/prilocaine cream |
| *Arm* | |
| Vachiramon et al., 2021 ^9^ | 1% lidocaine with adrenaline injection |
| Vachiramon et al., 2022 ^4^ | Forced air using Cryo 6 (Zimmer MedizinSysteme GmbH) at airflow level 5 (917 L/min, −28°C) starting and ending at the same time as the MFU- V procedure.and/or topical anesthetic cream (2.5%/2.5% lidocaine- prilocaine; Galentic Pharma), with an occlusive dressing |
| *Décolletage* | |
| Fabi et al., 2013 ^45^ | diazepam (5-10 mg), 200 mg of ibuprofen, or 500 mg of acetaminophen |
| Fabi et al., 2015 ^46^ | Oral diazepam, ibuprofen, or acetaminophen could be administered at the discretion of the investigator |
| *Abdomen* | |
| Lin et al., 2020 ^47^ | 1 g of paracetamol, cold packs and topical local anesthesia using lignocaine 2% gel or local anesthesia with lignocaine and bupivacaine |
| Vachiramon et al., 2020 ^10^ | 1% lidocaine with adrenaline injection |
| *Knee* | |
| Gold et al., 2014 ^48^ | Diazepam 5–10 mg or hydrocodone/APAP 7.5/750 mg |

**Supplemental Table 4.** Risk of bias according to Risk Of Bias In Non-randomized Studies (ROBINS-I).

| **Author, year** | **Pre-intervention** | | **At intervention** | **Post-intervention** | | | |
| --- | --- | --- | --- | --- | --- | --- | --- |
|  | **Bias due to/in** | | **Bias in** | **Bias due to/in** | | | |
|  | **Confounding** | **Selection of participants** | **Classification of intervention** | **Deviations from intended interventions** | **Missing data** | **Measurement of outcomes** | **Selection of the reported result** |
| Rokhsar et al., 2015 ^42^ | Serious Risk | Low risk | Low Risk | No information | Low Risk | Serious Risk | Low Risk |
| Goldberg et al., 2014 ^43^ | Serious Risk | Low risk | Low Risk | No information | Moderate Risk | Serious Risk | Low Risk |
| Fabi et al., 2014 ^26^ | Serious Risk | Low Risk | Low Risk | No information | Low Risk | Serious Risk | Low Risk |
| Lim et al., 2023 ^18^ | Serious Risk | Low risk | Low Risk | No information | Low Risk | Serious Risk | Low Risk |
| Shome et al., 2019 ^27^ | Serious Risk | Moderate Risk | Low Risk | Low Risk | Low Risk | Serious Risk | Low Risk |
| Gold et al., 2014 ^48^ | Serious Risk | Low Risk | Low Risk | Low Risk | Low Risk | Serious Risk | Low Risk |
| Oni et al., 2014 ^22^ | Serious Risk | Low risk | Low Risk | Low Risk | Low Risk | Serious Risk | Low Risk |
| Lee et al., 2015 ^23^ | Serious Risk | Low Risk | Low Risk | Low Risk | Low Risk | Low Risk | Low Rik |
| Fabi et al., 2013 ^45^ | Serious Risk | Low risk | Low Risk | Low Risk | Low Risk | Serious risk | Low Rik |
| Werschler et al., 2016 ^32^ | Serious Risk | Low Risk | Low Risk | Low Risk | Low Risk | Serious risk | Low Risk |
| Lin et al., ^47^ | Serious Risk | Low Risk | Low Risk | Low Risk | Low Risk | Serious risk | Low Risk |
| Fabi et al., 2015 ^46^ | Serious Risk | Low Risk | Low Risk | Low Risk | Low Risk | Serious risk | Low Risk |
| Araco et al., 2020 ^25^ | Serious Risk | Low Risk | Low Risk | Low Risk | Low Risk | Serious risk | Low Risk |
| Lu et al., 2017 ^33^ | Serious Risk | Low Risk | Low Risk | No information | Low Risk | Serious risk | Low Risk |
| Kerscher et al., 2019 ^31^ | Serious Risk | Low risk | Low Risk | Low Risk | Low Risk | Serious Risk | Low Risk |
| Sasaki et al., 2012 ^37^ | Serious Risk | Low risk | Low Risk | Low Risk | Low Risk | Serious Risk | Low Risk |
| Corduff et al., 2023 ^28^ | Serious Risk | Low risk | Low Risk | Low Risk | Moderate risk | Serious Risk | Low Risk |
| Montes et al., 2019 ^38^ | Serious risk | Low Risk | No information | Low Risk | Serious risk | Serious Risk | Low Risk |
| Fabi et al., 2020 ^44^ | Serious Risk | Low Risk | Low Risk | Low Risk | Low risk | Serious Risk | Low Risk |
| Baumann et al., 2016 ^36^ | Serious Risk | Low Risk | Low Risk | Low Risk | Low Risk | Serious Risk | Low Risk |
| Schlessinger et al., 2019 ^8^ | Serious Risk | Low Risk | Low Risk | Low Risk | Low Risk | Serious Risk | Low Risk |
| Jeon et al., 2021 ^20^ | Serious Risk | Low Risk | Low Risk | Low Risk | Low Risk | Serious Risk | Low Risk |
| Casabona et al., 2019 ^39^ | Serious Risk | Low Risk | Low Risk | Low risk | Low Risk | Serious Risk | Serious Risk |
| Maas et al., 2019 ^19^ | Serious Risk | Low Risk | Low Risk | Low Risk | Low Risk | Serious Risk | Low Risk |
| Harris et al., 2015 ^29^ | Serious Risk | Low Risk | Low Risk | Low Risk | Low Risk | Serious Risk | Low Risk |
| Wanitphakdeedecha et al., 2020 ^21^ | Serious Risk | Low Risk | Low Risk | Low Risk | Low Risk | Serious Risk | Low Risk |
| Yalici-Armagan et al., 2020 ^24^ | Serious Risk | Low Risk | Low Risk | Low Risk | Low Risk | Serious Risk | Low Risk |
| Chang et al., 2019 ^40^ | Serious Risk | Low Risk | Low Risk | Low Risk | Serious Risk | Serious Risk | Low Risk |
| Kerscher et al., 2019 ^30^ | Serious Risk | Serious Risk | Low Risk | Low Risk | Low Risk | Low Risk | Low Risk |
| Sasaki et al., 2017 ^41^ | Serious Risk | Low Risk | Low Risk | Low Risk | Low Risk | Serious Risk | Low Risk |
| Fusano et al., 2021 ^11^ | Serious Risk | Low Risk | Low Risk | Low Risk | Low Risk | Serious Risk | Low Risk |
| Tran et al., 2022 ^3^ | Serious Risk | Serious Risk | Low Risk | Low Risk | Low Risk | Serious Risk | Low Risk |

**Supplemental Table 5.** Risk of bias according to Cochrane Collaboration’s Tool Risk of Bias 2 (ROB2).

| **Author, year** | **Bias arising from** | | | | | **Overall score** |
| --- | --- | --- | --- | --- | --- | --- |
|  | **Randomization process** | **Intended interventions** | **Missing data** | **Outcome measurement** | **Selection of the reported result** |  |
| Vachiramon et al., 2021 ^13^ | High risk | Low risk | Low risk | Low risk | Low risk | Intermediate risk |
| Vachiramon et al., 2021 ^9^ | High risk | High risk | Some concern | Some concern | Low risk | High risk |
| Vachiramon et al., 2023 ^4^ | Low risk | High risk | Low concern | High risk | Low risk | High risk |
| Polacco et al., 2020 ^5^ | High risk | High risk | Some concern | High risk | Low risk | High risk |
| Vachiramon et al., 2023 ^6^ | Low risk | Low risk | Some concern | Low risk | Low risk | Low risk |
| Palm et al., 2018 ^7^ | High risk | High risk | Some concern | High risk | Low risk | High risk |
| Yusova et al., 2019 ^12^ | High risk | Some concern | Some concern | Some concern | Low risk | High risk |
| Vachiramon et al., 2020 ^10^ | Low risk | Low risk | Some concern | Low risk | Low risk | Low risk |
| Jones et al., 2017 ^35^ | High risk | High risk | Some concern | High concern | Low risk | High risk |
| Alhaddad et al., 2019 ^34^ | High risk | High risk | Some concern | High concern | Low risk | High risk |

**Supplemental Table 6.** Summary of aesthetic improvement scores and patients satisfaction following MFU-V treatment.

| **Author, year** | **Treated region** | **Reported outcomes/ Assessment methods** | **Summary of finings** |
| --- | --- | --- | --- |
| ***Facial regions*** | | | |
| Wanitphakdeedecha et al., 2020 ^21^ | Upper face | - **Improvement score-investigators**/quartile grading scale - **Improvement score- patients**/ quartile grading scale | - As early as 1-week follow-up, 64.7% had minimal improvement (0%-25%) when compared to the baseline. At 1-month follow-up, majority (82.4%) still had minimal improvement, which was consistent until the 3-month follow-up (67.6%). However, at 6-month follow-up, most (51.9%) showed no improvement (0%) when compared to baseline. - As early as 1-week follow-up, majority (46.4%) reported minimal improvement, which continued to increase at 1-month (46.4% moderate improvement) and 3-month follow-up (53.6% marked improvement). However, on the 6-month follow-up, there was a decline in the improvement score wherein majority (38.1%) had moderate improvement |
| Oni et al, 2014 ^22^ | Lower face | - **Improvement score-Investigators**/3-point scale (Improvement, No Change, Worse) - **Improvement score- patients**/ Improved; not improved | - 58.1% of patients were evaluated as improved, 17.2% no change, and 24.7% worse results at day 90. - 65.6% of patients reported themselves as improved and 34.4% as no improved at day 90. |
| Araco et al., 2020 ^25^ | Full face treatment except for the upper one-third | - **Improvement score-Investigators**/grading before-after images from 1-20, total scoring from 20-100. - **Patient satisfaction**/10 point scale. | - In total patients were scored 80.48 ± 4.2 points at day 90. - 7.28 ± 1.16 point of satisfaction at day 90. |
| Vachiramon et al., 2021 ^13^ | Cheeck | **Patient satisfaction**/ visual analog scale on a scale from 0 (not satisfied) to 10 (10 extremely satisfied). | At month 1, mean ± SD, 4.43±3.12 of satisfaction; at month 2, 5.53±2.82; at month 3, 6.42±2.60; at month 6, 6.84±2.96 |
| Yalici-Armagan et al., 2020 ^24^ | Lower face | - **Improvement score-Investigators**/ GAIS - **Improvement score-Patients**/ GAIS | Investigators: 15 subjects (62.5%) were assessed as no change. Among 5 subjects (20.9%) who had any improvement, 4 of them (16.7%) were assessed as mildly improved and 1 subject (4.2%) was assessed moderately improved. Four subjects (16.7%) were scored as worsening.  Patients: 11 subjects (45.9%) reported an improvement whereas 9 subjects (37.5%) reported no change. Two (8.3%) subjects were rated themselves as mildly improved, 8 (33.4%) subjects moderately improved and 1 subject (4.2%) very much improved. No subject reported worsening. |
| ***Facial and Neck regions*** | | | |
| Shome et al,. 2019 ^27^ | Lower half of the face and neck region | - **Improvement Score- Investigators**/ 2 independent investigators using the Investigator Assessment Scale on standardized photographs - **Improvement Score- Patients**/a subjective Assessment Scale | - After 2 months, mean ± SD of improvement, 1.6 ± 0.76; After 6 months, 2.64 ± 0.48; After 1 year, 2.60 ± 0.50. - After 2 months, mean ± SD of improvement, 1.52 ± 0.65; After 6 months, 2.56 ± 0.50; After 1 year, 2.50 ± 0.48. |
| Kerscher et al., 2019 ^30^ | Submental region and contour the jawline | Improvement score-investigators/ GAIS | At week 12, based on frontal view, 50% of the participants were evaluated as improved and the half as much improved. The same results were reported in the side view. |
| Fabi et al., 2014 ^26^ | Face and upper neck | - **Improvement score-Investigators**/ GAIS - **Improvement score-Patients**/ GAIS - **Patient Satisfaction**/ 4 point scale (Very Satisfied, satisfied, dissatisfied, very dissatisfied) | Investigators: At day 90, 13 (81.3) patients were scored as any improvement and while at day 90, 35 (77.7) were scored as any improvement.  Patients: At day 90, 12 (75) patients self-scored as any improvement and while at day 90, 35 (77.8) were scored as any improvement.  At 90 days after treatment, 62.5% of subjects who had completed the questionnaire indicated that they were very satisfied (25%) or satisfied (37.5%) with the treatment results. At 180 days after treatment, 60% of subjects continued to be very satisfied (11.1%) or satisfied (48.9%) with their treatment. |
| ***Neck*** | | | |
| Jones et al, 2017 ^35^ | Neck | **Patient Satisfaction**/ 5 point scale (0 not satisfied and 4 extremely satisfied) | Satisfaction mean (SD) of 2.1 (1.88) t day 180. |
| ***Abdomen*** | | | |
| Vachiramon et al., 2020 ^10^ | Lower abdomen_ S***ingle‐plane*** | - **Improvement Score- Investigators**/ 0 (no improvement) to 10 (significant improvement) - **Improvement score- Patients** | - Mean improvement scores were 3.03 (±1.26), 3.43 (±1.35), and 2.18 (±0.86) at 1‐,3‐, and 6‐month follow‐up, respectively. - Mean improvement scores were 4.69 (±2.30), 5.26 (±2.32), and 3.46 (±3.18) at 1‐,3‐ and 6‐month follow‐ups, respectively. |
|  | Lower abdomen _ ***Dual‐plane*** | - **Improvement Score- Investigators**/ 0 (no improvement) to 10 (significant improvement) - **Improvement score- Patients** | - Mean improvement scores were 3.11 (±1.23), 3.39 (±1.34), and 2.02 (±0.79) at 1‐,3‐, and 6‐month follow‐up, respectively. - Mean improvement scores at 1‐,3‐, and 6‐month follow‐ups were 4.61 (±2.29), 5.14 (±2.40), and 3.44 (±3.24). |
| ***Arm*** | | | |
| Vachiramon et al., 2021 ^9^ | Upper Arm (posterior arm)_ S***ingle‐plane*** | - **Improvement score-Investigators**/NM - **Improvement score-Patients**/NM | - 2.22 (±1.05) of improvement at 1 month, 2.70 (±1.32) at 3 months, and 1.61 (±0.79) at 6 months. - 3.63 (±2.63), 3.69 (±2.83), and 1.90 (±2.51) of improvement at 1, 3, and 6 months, respectively. |
|  | Upper Arm (posterior arm)_***Dual‐plane*** | - **Improvement score-investigators**/NM - **Improvement score-Patients**/NM | - 2.00 (±1.00) of improvement at 1 month, 2.56 (±1.05) at 3 months, and 1.39 (±0.54) at 6 months. - 3.5 (±2.57), 3.22 (±2.59), and 1.78 (±2.48) of improvement at 1, 3, and 6 months follow‐up, respectively |
| ***Décolletage*** | | | |
| Fabi et al., 2020 ^44^ | Décolleté area | **Patients satisfaction**/ 7 point scale 0 (completely dissatisfied) to 6 (completely satisfied) | At day 360, mean (SD) subject satisfaction scores had increased from 2.9 (1.8) on day 180 to 3.9 (1.8) on day 360. |
| ***Combination of several body parts*** | | | |
| Sasaki et al., 2017 ^41^ | Forehead, periorbitum, face, neck, and décolleté | **Physician Global Aesthetic Improvement** / PGAIS | Mean of improvement after utilization of two transducers: Forehead: 3.0 ± 2.2; Periorbitum: 3.0 ± 2.1; Midface: NA; Neck: NA; Décolletage: NA.  Mean of improvement after utilization of three transducers: Forehead: NA; Periorbitum: NA; Midface: 3.0 ± 2.4; Neck: 3.0 ± 2.2; Décolletage: 3.0 ± 0.4. |
| Casabona et al., 2019 ^39^ | Breasts, buttocks, thighs, or abdomen | **Patient satisfaction**/ 4 point scale (5 very satisfied to 1 unsatisfied) | Mean Patient Satisfaction Scale score increased from 3.75 (±0.44) to 4.70 (±0.47) at 90 days (p<0.001). |
| Montes et al., 2019 ^38^ | Lower face, submentum, neck  Eyes, décolleté | - **Improvement score-Patients**/Online survey- 13 questions related to the treatment process and post-treatment outcomes. - **Patient satisfaction**/ 5 point scale (Very satisfied to very dissatisfied) | A large number of patients (41%) reported their treatment outcome met or exceeded their expectation, and nearly all (92%) anticipated that their treatment results would last for 1 year.  Very satisfied; 9 patient self-scored as very satisfied, 17 as satisfied, 15 as neutral, 9 as dissatisfied, and 3 as very dissatisfied. |
| Chang et al., 2019 ^40^ | Full face, Full face/neck, Lower face, Lower face/neck, Jawline, Brow, Neck, Chest, Body. | **Patient satisfaction/** 4 point scale (disappointed, neutral, satisfied, extremely satisfied) | At month 4after treatment 22 (27.2%) of patients were disappointed, 16 (19.8%) neutral, 31 (38.3%) satisfied, and 12 (14.8%) extremely satisfied. |

**Supplemental Table 7.** Summary of pain scores following MFU-V treatment.

| **Author, year** | **Summary of finings** |
| --- | --- |
| ***Facial regions*** | |
| Lim et al., 2023 ^18^ | The procedure was well tolerated with only mild-to-moderate pain reported in 37 of 40 treatments (92.5%). Using a 10-point VAS, patients reported a median pain score of 3, with 24 treatments giving a VAS pain rating of 0–3 (mild), 13 treatments giving a rating of 4–7 (moderate) and 3 treatments giving a rating of 8–10 (severe) |
| Maas et al., 2019 ^19^ | The mean pain scores during treatment with the 7 MHz/3.0 mm transducer were 4.2, 5.2, and 5.0 for treatments 1, 2, and 3, respectively. For the 10 MHz/1.5 mm transducer, the mean pain scores were 4.9, 6.2, and 5.9 for treatments 1, 2, and 3, respectively. Scores for both transducers indicate moderate pain severity. |
| Jeon et al., 2020 ^20^ | Pain was reported by approximately half of the patients which was mild in severity. |
| Yalici-Armagan et al., 2020 ^24^ | Patients experienced only minimal pain during the treatment session. No patient reported severe pain requiring additional pain relief with analgesia or sedation. None dropped out of the study due to intolerable pain or side effects. |
| Vachiramon et al., 2021 ^13^ | Pain was reported by some of the patients. |
| ***Facial and Neck regions*** | |
| Shome et al., 2019 ^27^ | All patients experienced mild-to-moderate pain during the treatment session. Thirty-two percent patients faced mild pain; 48% faced moderate pain, and 20% faced severe pain during the procedure. |
| Kerscher et al., 2019 ^31^ | Self-assessment of pain during treatment of the lower face and submental region revealed minimal to moderate pain (VAS score, 1–5) for 15 cases, with 7 patients reporting minimal pain and 6 patients reporting moderate pain. For all patients, pain diminished shortly after treatment. |
| Palm et al., 2018 ^7^ | Similar mean pain scores of 5.6 were observed for 23/7 and lidocaine 4%/benzocaine 20% during treatment. |
| ***Neck*** |  |
| ***Combination of several body parts*** | |
| Sasaki et al., 2012 ^37^ | Periorbitum (n=19): Mild (Scores 1 - 4): 15; Moderate (5 - 8): 2; Severe (9 - 10): 2  Décolletage (n=5): Mild (Scores 1 - 4): 1; Moderate (5 - 8): 2; Severe (9 - 10): 2  Brachium (n=44): Mild (Scores 1 - 4): 10; Moderate (5 - 8): 21; Severe (9 - 10): 13  Hand (n=1): Mild (Scores 1 - 4): 0; Moderate (5 - 8): 1; Severe (9 - 10): 0;  Periumbilicus (n=6): Mild (Scores 1 - 4): 0; Moderate (5 - 8): 5; Severe (9 - 10): 1  Buttock (n=2): Mild (Scores 1 - 4): 0; Moderate (5 - 8): 1; Severe (9 - 10): 0  Inner Thigh (n=1): Mild (Scores 1 - 4): 0; Moderate (5 - 8): 1; Severe (9 - 10): 0  Knee(n=4): Mild (Scores 1 - 4): 0; Moderate (5 - 8): 3; Severe (9 - 10): 1 |
| Chang et al., 2019 ^40^ | Pain scores were varied, from mild (30%), moderate (35%), and significant (25%) |
| Sasaki e al., 2017 ^41^ | Although all participants experienced pain during treatments, none of them required local anesthesia or nerve blocks to complete treatment protocols.  About 5% (14 patients) of off-study patients, especially those treated at 3 levels, experienced low grade pain or hypersensitivity for more than a week after treatment. |
| ***Elbow*** | |
| Rokhsar et al., 2015 ^42^ | The mean pain score during treatment with the 4 MHz, 4.5 mm transducer was 5.7, and during treatment with the 7 MHz, 3.0 mm, transducer, the mean pain score was 5.0. |
| ***Arm*** | |
| Vachiramon et al., 2021 ^9^ | The median pain scores were 2.6 (0–8) for single plane treatment and 1.9 (0–8) for dual‐plane treatment |
| ***Abdomen*** | |
| Lin et al., 20202 ^47^ | These patients reported a mean pain score of 7.6. |
| Vachiramon et al., 2020 ^10^ | The median pain scores were 5.25 (2–10) for single plane treatment and 4.29 (0–8) for dual plane treatment. |


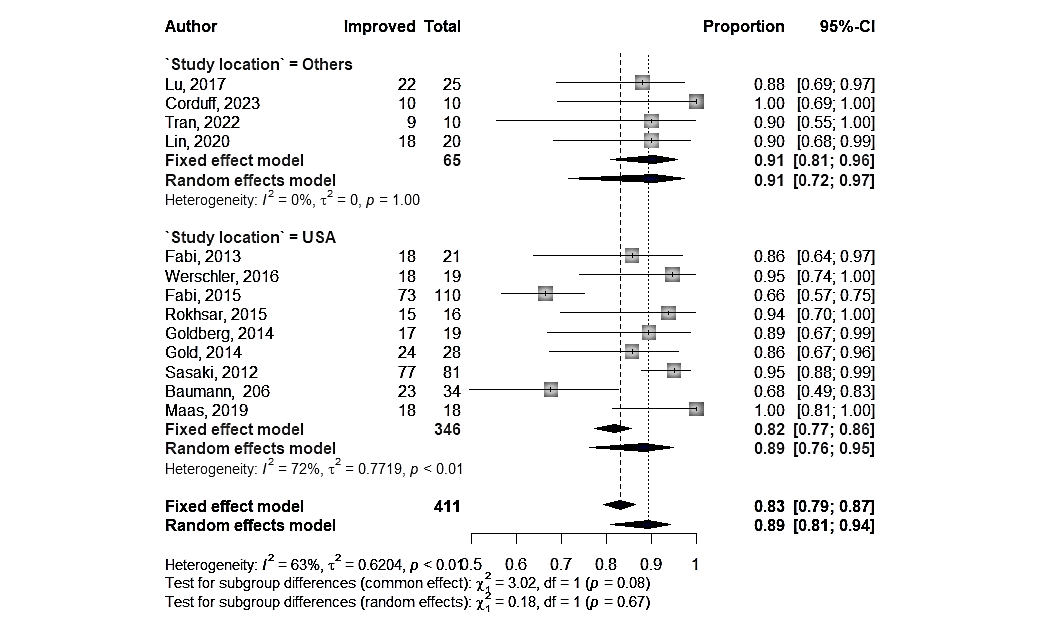


A


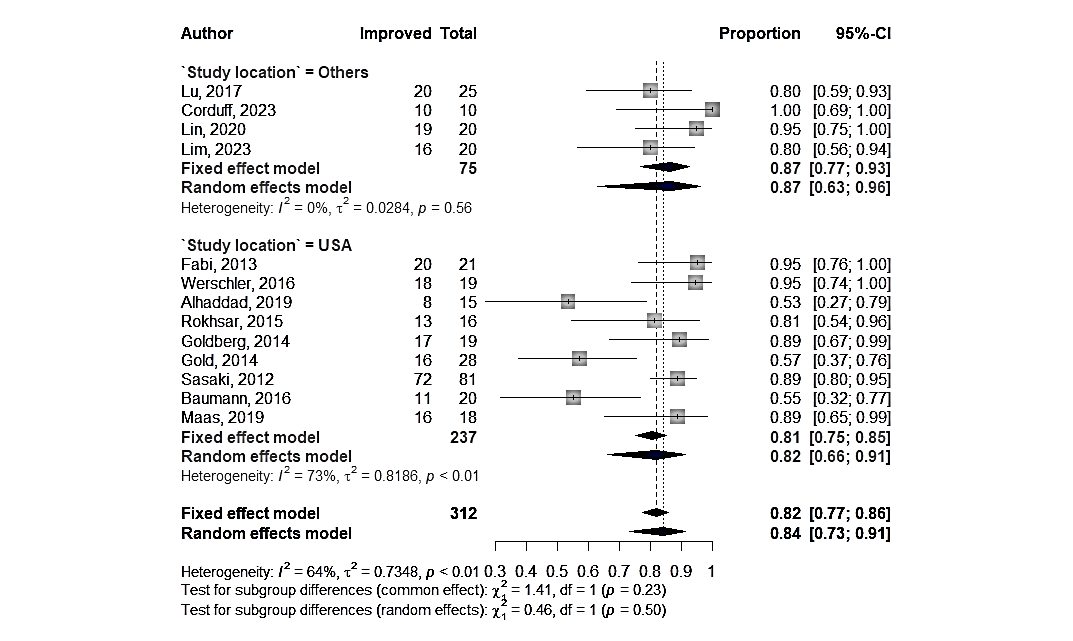


B


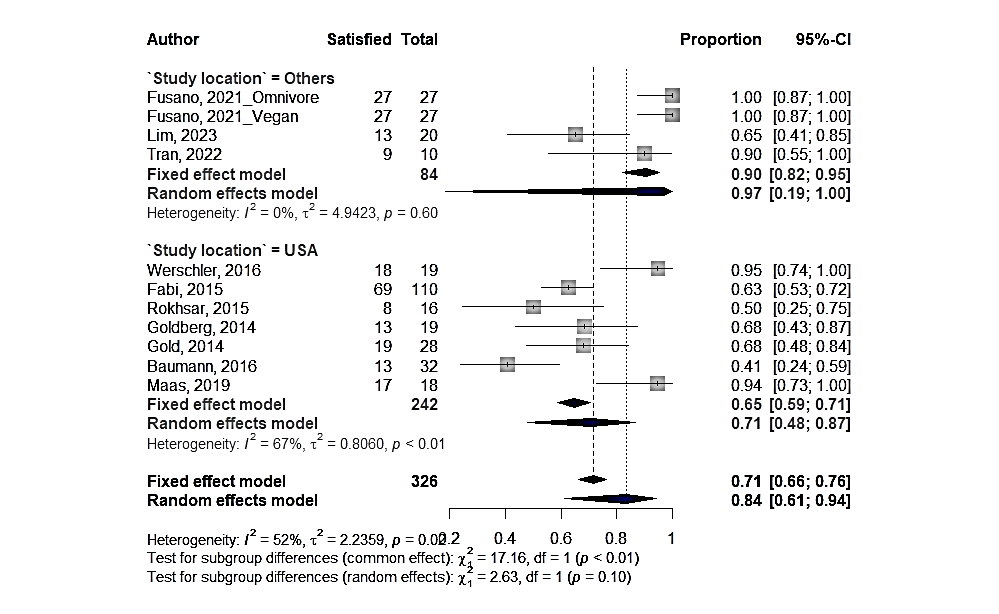


C


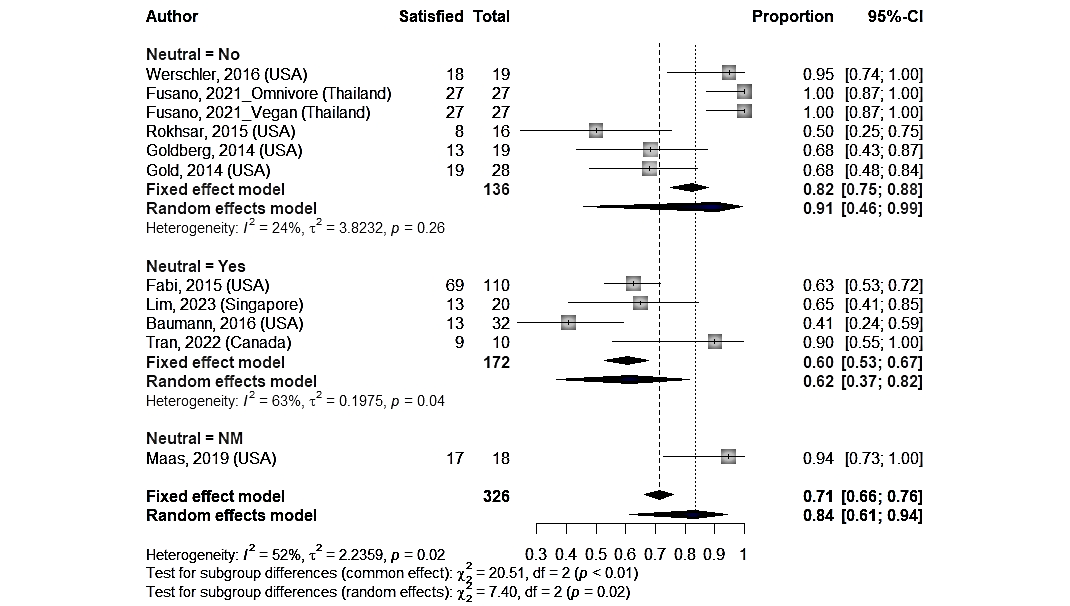


D

**Supplemental Figure 1.** Summary proportions and pooled estimates of analysis of **A)** Investigators global aesthetic improvement; **B)** Patients global aesthetic improvement; **C)** Patients satisfaction based on study location and **D)** Patients satisfaction according to the presence of ‘Neutral’ among the potential responses.


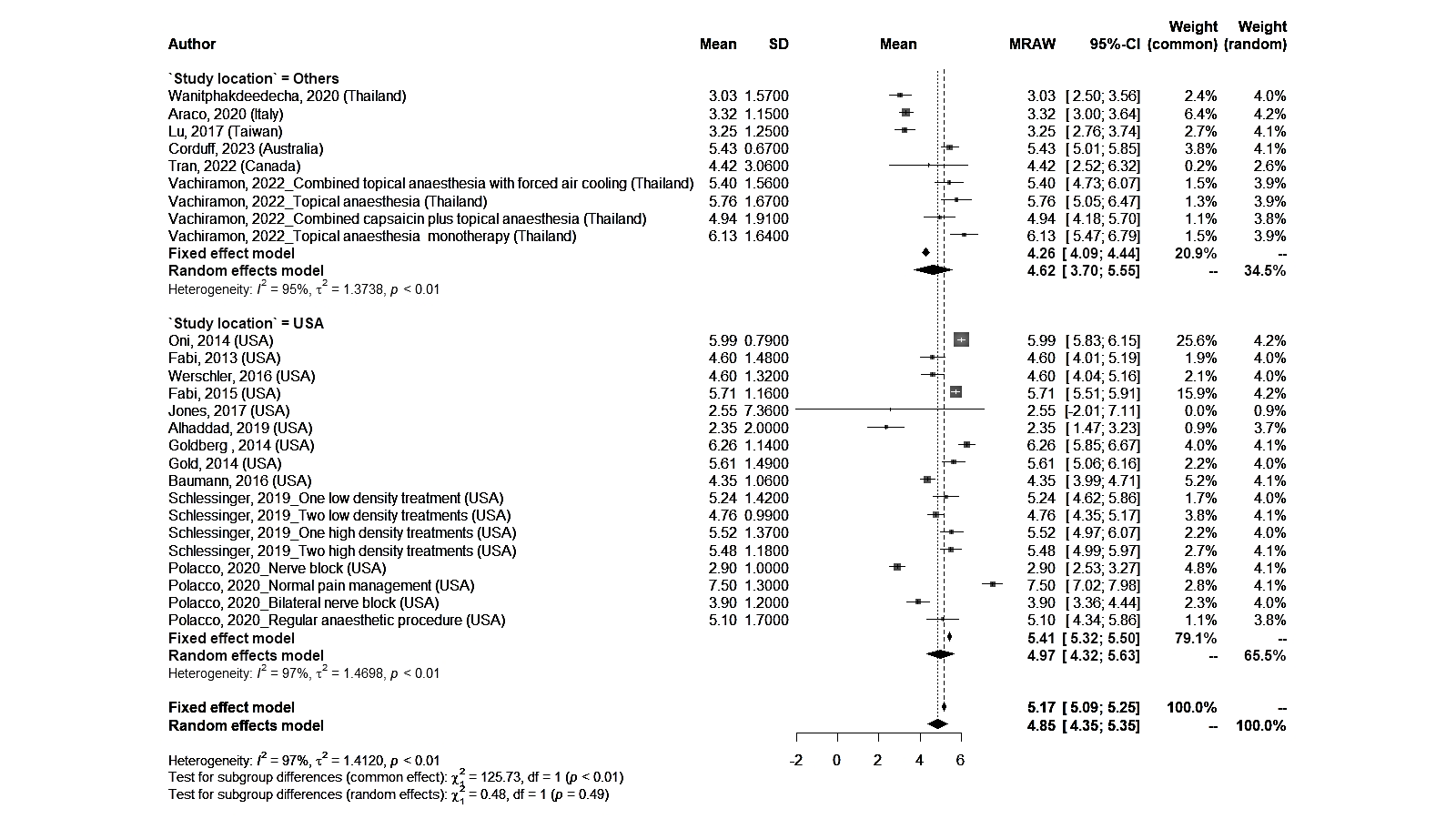


**Supplemental Figure 2.** Summary proportions and pooled estimates of pain score following MFU-V treatment based on study location.

**Supplemental Table 8.** Findings of meta regression, follow-up time (day) as predictor variable.

| **Outcome** | **No. of effect estimates** | **I^2^ (%)** | **Beta coefficient** | **95% Confidence interval** | **p-value** |
| --- | --- | --- | --- | --- | --- |
| **Investigator global aesthetic improvement** | 13 | 66.57 | 0.004 | -0.001, 0.02 | 0.5 |
| **Patients global aesthetic improvement** | 13 | 66.07 | 0.008 | -0.008, 0.02 | 0.28 |
| **Patients satisfaction** | 11 | 90.15 | 0.006 | -0.01, 0.02 | 0.5 |

**Supplemental Figure 3.** Funnel plots and Egger’s test p-values of the included studies in the meta-analysis of **A)** Investigators global aesthetic improvement; **B)** Patients global aesthetic improvement; **C)** Patients satisfaction; **D)** Pain score following MFU-V treatment


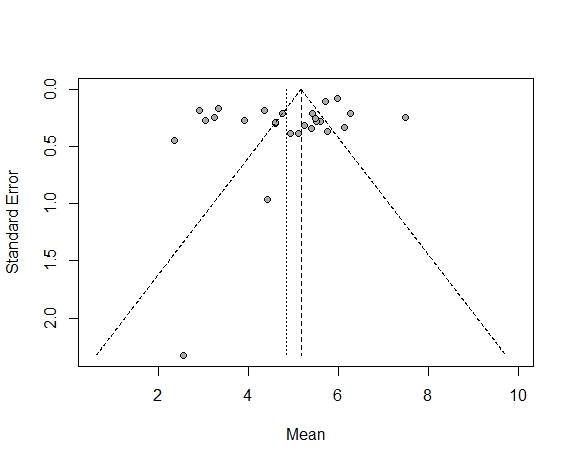


D

Egger’s test, p-value = 0.11


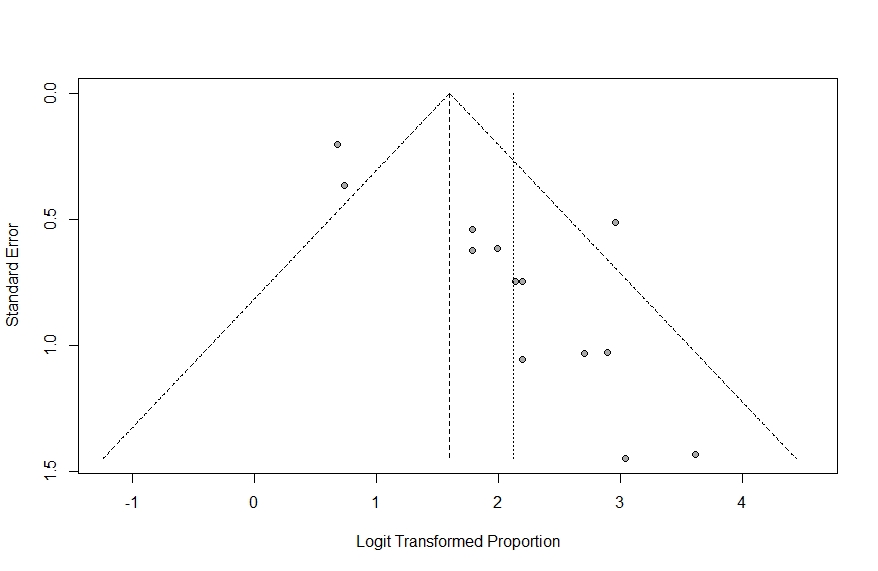


Egger’s test, p-value = 0.0002

A


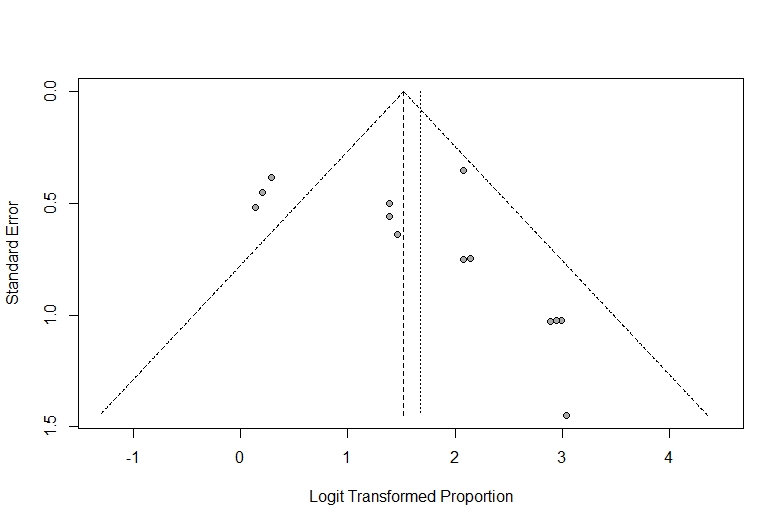


B

Egger’s test, p-value = 0.06


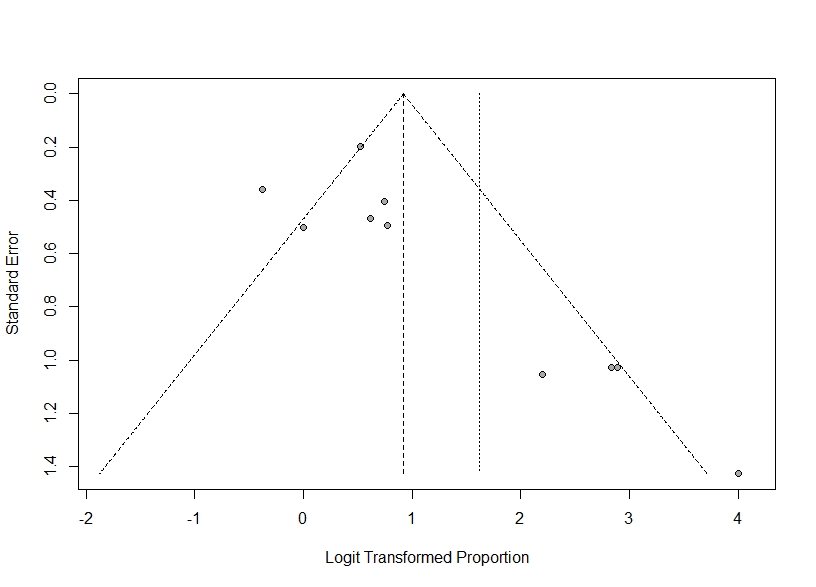


C

Egger’s test, p-value = 0.01


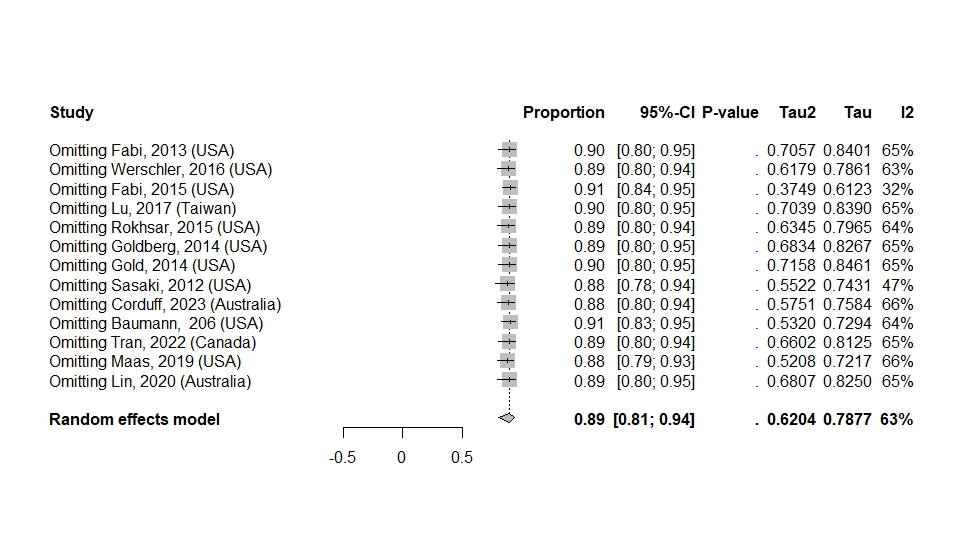


A


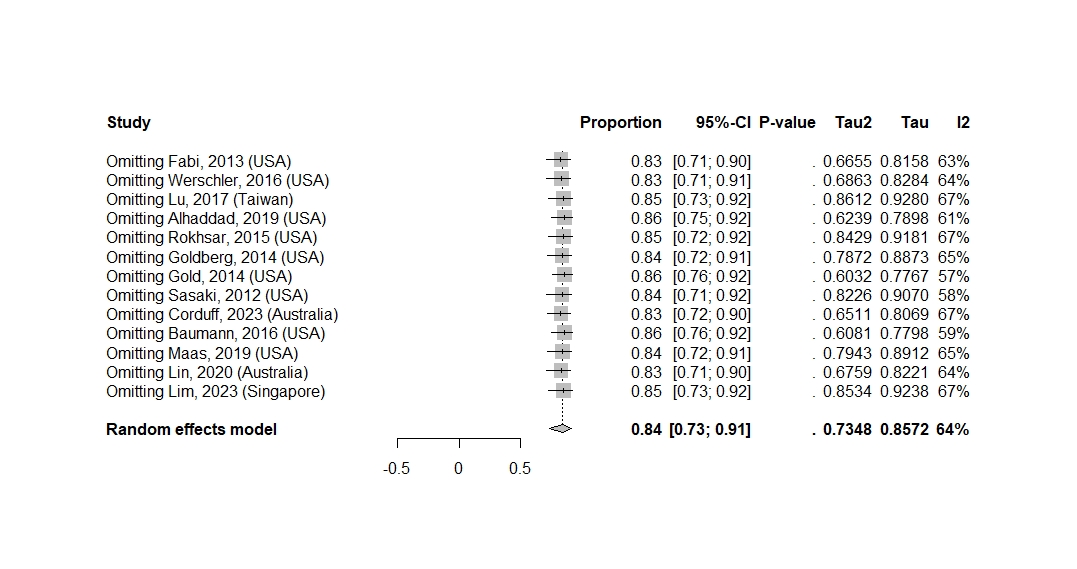


B


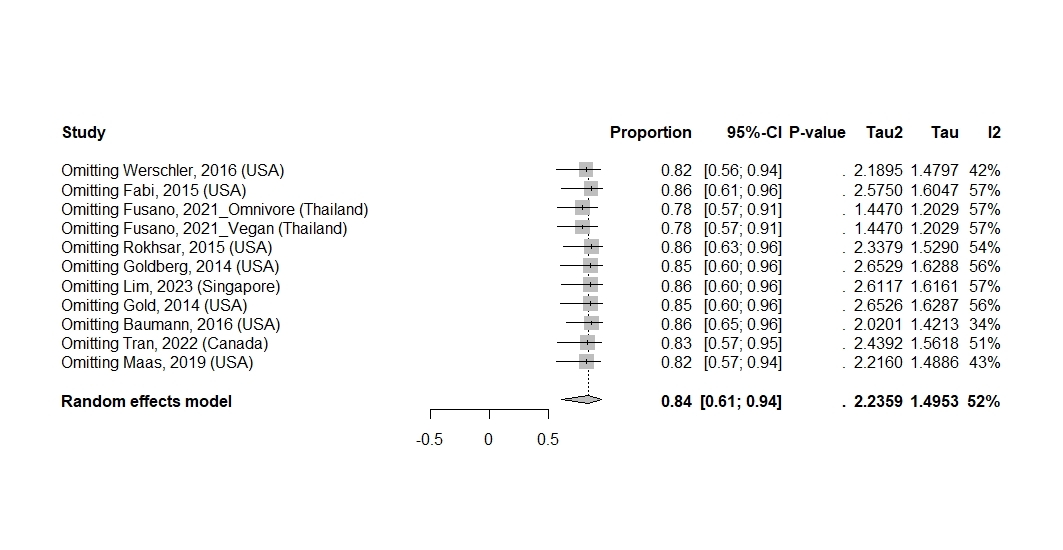


C

**Supplemental Figure 4**. Leave-one-out analysis of the included studies in the meta-analysis of **A)** Investigators global aesthetic improvement; **B)** Patients global aesthetic improvement; **C)** Patients satisfaction; **D)** Pain score following MFU-V treatment


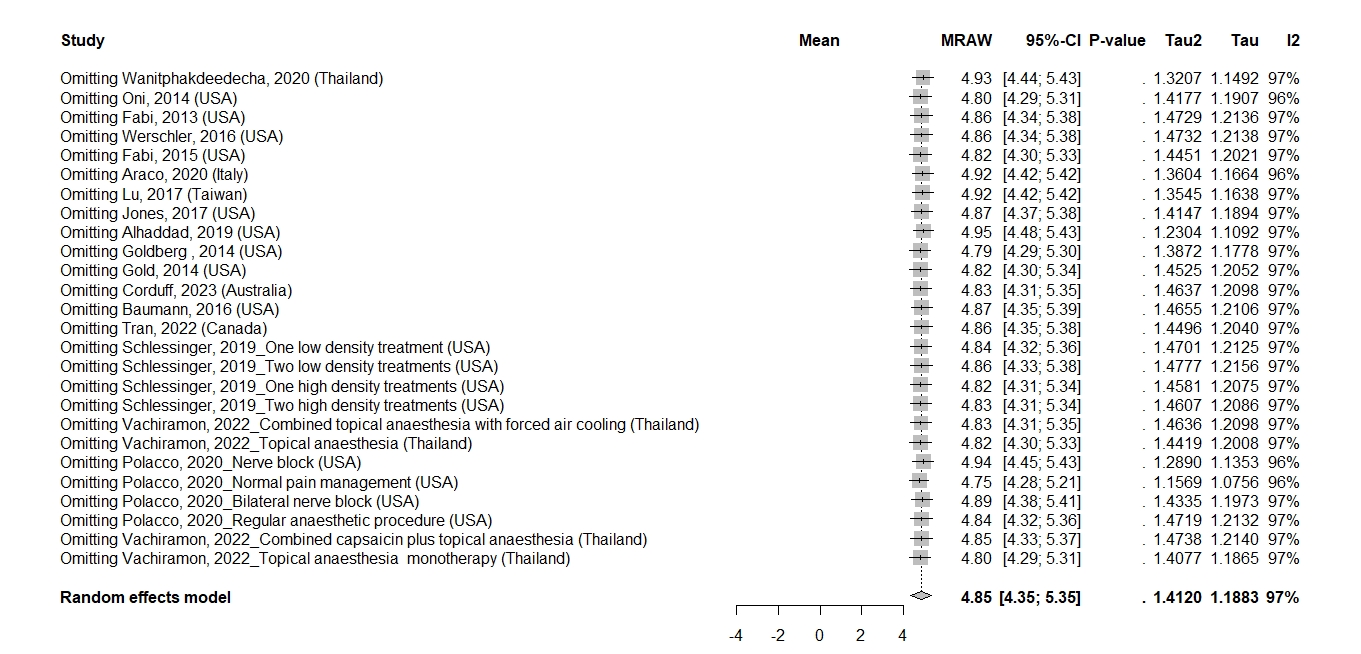


D

**Supplemental Table 9**. Summary of skin quality findings following MFU-V treatment.

| **Author, year** | **Treated region** | **Reported outcomes/ Assessment methods** | **Summary of finings** |
| --- | --- | --- | --- |
| ***Facial regions*** | | | |
| Yusova et al., 2019 ^12^ | Lower third of the face and the submandibular space | **Thickness**/ DUB Skinscanne (μm) | Thickness of epidermis (Mean ± SD): 96.6±0.68 at baseline and 107.6±0.236 at month 6 after treatment.  Thickness of dermis (Mean ± SD): 1338.9±0.376 at baseline and 1490.7± 0.471 at month 6 after treatment. |
| Wanitphakdeedecha et al., 20202 ^21^ | Upper face | **Wrinkles**, **skin roughness**/ Antera 3D | Wrinkles (Mean ± SD): 15.86±3.2 at baseline, 14.83±3.35 at week 1, 15.51± 3.38 at month 1, 15.38± 4.15 at month 3, and 14.97± 3.79 at month 6 after treatment (vs baseline: p = 0.002, p =1.0, p =0.70, p =0.01).  Skin roughness (Mean ± SD): 16.07±4.38 at baseline, 15.21±4.35 at week 1, 15.77±4.52 at month 1, 15.57±4.91 at month 3, and 15.63±4.28 at month 6 after treatment (vs baseline: p = 0.92, p =1.0, p =1.0, p =0.004). |
| Lee et al., 2015 ^23^ | Face | **Laxity** and **sagging**/ Graded from 1 to 6 using Automatic Skin Diagnosis | Median grade of laxity and sagging: 5 (4-5) at baseline, 3 (2-3) at month 2 after treatment and 3 (3-4) at month 4 (month 4 vs 2; p <0.01) |
| Vachiramon et al., 2021 ^13^ | Cheeck | **Pore/** Antera 3D and Physician assessment | A lower mean pore volume were observed after 4 months. |
| ***Facial and Neck regions*** | | | |
| Werschler et al., 2016 ^32^ | Lower face and neck | **Wrinkle**, **sagging**, **evenness**, **smoothness**/ Questionnaire (Lines/wrinkles, Less Sagging, More Even Skin Tone/Color, Smoother Skin Texture, No Improvement) | Presence of lines/wrinkles: 50% at day 90, 37% at day 180, 58% 1 year after treatment.  Less Sagging: 90% at day 90, 84% at day 180, 79% 1 year after treatment.  More Even Skin Tone/Color: 10% at day 90, 21% at day 180, 0% 1 year after treatment.  Smoother Skin Texture: 45% at day 90, 47% at day 180, 47% 1 year after treatment.  No Improvement: 0% at day 90, 5% at day 180, 5% 1 year after treatment. |
| Lu et al., 2017 ^21^ | Face and neck | **Wrinkle**, **texture, pores/** Skin complexion analysis and 3-dimensional imaging system | Mean wrinkles score: 50.24 at baseline, 42.68 at day 90 and 45.52 at day 180 after treatment (vs baseline; p=0.02, p=0.12).  Mean texture score: 2,085.56 at baseline, 2061.12 at day 90 and 2282.52 at day 180 after treatment (vs baseline; p=0.86; p=0.12).  Mean pores score: 768.88 at baseline, 711.68 at day 90 and 777.96 at day 180 after treatment (vs baseline; p=0.10, p=0.71). |
| Alhaddad et al., 2019 ^34^ | Face  Upper neck | **Laxity**/ The Fasil Face and Neck Laxity Grading Scale | **MFU-V (Mean ± SD scores):**  Eyelid: 3.15±0.8 before treatment; 2.47±1.3 at day 30; 2.61±1.3 at day 90 and 2.67±1.2 at day 180 after treatment (vs baseline: p=0.053, p=0.14, p=0.16).  Cheek bone roundness: 3.25±0.9 before treatment; 1.89±0.9 at day 30; 2.22±1.3 at day 90 and 2.40±1.3 at day 180 after treatment (vs baseline: p<0.01, p<0.01, p=0.025).  Melolabial folds: 3.15±0.9 before treatment; 2.10±1.1 at day 30; 2.06±1.0 at day 90 and 2.27±1.0 at day 180 after treatment (vs baseline: p<0.01, p<0.01, p<0.01).  Jowls: 3.1±0.8 before treatment; 2.05±1.1 at day 30; 1.78±1.1 at day 90 and 2.27±1.3 at day 180 after treatment (vs baseline: p<0.01, p<0.01, p=0.027).  Horizontal folds: 2.85±0.8 before treatment; 2.0±1.3 at day 30; 2.2±1.35 at day 90 and 2.47±1.5 at day 180 after treatment (vs baseline: p=0.02 , p=0.08, p=0.3).  Platysmal bands: 2.6±0.8 before treatment; 1.47±1.2 at day 30; 1.61±1.0 at day 90 and 1.8±1.5 at day 180 after treatment (vs baseline: p<0.01 , p<0.01 , p=0.048).  **MRF (Mean ± SD scores):**  Eyelid: 3.15±0.8 before treatment; 2.47±1.3 at day 30; 2.61±1.3 at day 90 and 2.60±1.3 at day 180 after treatment (vs baseline: p=0.064, p=0.14, p=0.13).  Cheek bone roundness: 3.25±0.9 before treatment; 1.74±0.9 at day 30; 2.0±1.1 at day 90 and 2.33±1.2 at day 180 after treatment (vs baseline: p<0.01, p<0.01, p=0.01).  Melolabial folds: 3.15±0.9 before treatment; 1.89±0.7 at day 30; 2.0±1.0 at day 90 and 2.3±1.0 at day 180 after treatment (vs baseline: p=<0.01, p<0.01, p=0.02).  Jowls: 3.1±0.8 before treatment; 1.95±0.9 at day 30; 1.78±0.8 at day 90 and 2.2±1.4 at day 180 after treatment (vs baseline: p<0.01, p<0.01, p=0.025).  Horizontal folds: 2.85±0.8 before treatment; 2.37±1.2 at day 30; 2.3±1.3 at day 90 and 2.5±1.5 at day 180 after treatment (vs baseline: p=0.08 , p=0.09, p=0.3).  Platysmal bands: 2.6±0.8 before treatment; 1.58±1.2 at day 30; 1.83±1.1 at day 90 and 2.0±1.4 at day 180 after treatment (vs baseline: p<0.01 , p=0.01 , p=0.08). |
| Kerscher et al., 2019 ^30^ | Submental region and contour the jawline | **Thickness**, **firmness**/ Cutometry | Skin thickness: Mean of 1651 µmol at baseline, 1822.278 at week 4 and 1776 after 12 weeks.  Skin Firmness: Mean of 0.079 mmol at baseline, 0.113 at week 4 and 0.077 after 12 weeks. |
| Kerscher et al., 2019 ^31^ | Lower face and submental  region | **Elasticity**/ Cutometry | A single MFU-V treatment significantly increased the net skin elasticity at 12 and 24 weeks compared with the baseline (p<0.05). After 4 weeks, the net elasticity was significantly lower than that at baseline, suggesting physiologic restructuring of collagen tissue. Skin gross elasticity also showed decreased values at week 4, but significantly increased values at weeks 12 and 24 |
| Fusano et al., 2021 ^11^ | Lower face (cheeks, submental area and mandibular lines), neck _ O***mnivore patients*** | **Laxity**/ Facial Laxity Rating (FLR) | Skin laxity (Median (range)): In lower face at baseline 5 (3-7), 3 (1-6) at month 3 and 3 (1-6) at month 6 after treatment.  Skin laxity (Median (range)): In neck at baseline 4 (2-6), 3 (0-5) at month 3 and 2 (0-5) at month 6 after treatment. |
|  | Lower face (cheeks, submental area and mandibular lines), neck _ ***Vegan patients*** | **Laxity**/ Facial Laxity Rating (FLR) | Skin laxity (Median (range)): In lower face at baseline 5 (3-7), 3 (1-6) at month 3 and 3 (1-6) at month 6 after treatment.  Skin laxity (Median (range)): In neck at baseline 4 (2-6), 3 (2-6) at month 3 and 4 (2-6) at month 6 after treatment. |
| Fabi et al., 2014 ^26^ | Face and upper neck | **Wrinkle**, **sagging**, **evenness** / Questionnaire (Presence of lines and wrinkles; Less Skin Laxity (Sagging); Tighter/Lifted Skin; More Even Skin Tone (Color); Smoother Skin Texture; Overall Skin Health) | Presence of lines/wrinkles: 5 (31.3%) at day 90 and 14 (31.1%) at day 180 after treatment.  Less Skin Laxity (Sagging): 7 (43.8%) at day 90 and 22 (48.9%) at day 180 after treatment.  Tighter, Lifted Skin: 10 (62.5%) at day 90 and 27 (60.0%) at day 180 after treatment.  More Even Skin Tone (Color): 6 (37.5%) at day 90 and 10 (22.2%) at day 180 after treatment.  Smoother Skin Texture: 6 (37.5%) at day 90 and 18 (40.0%) at day 180 after treatment.  Overall Skin Health: 6 (37.5%) at day 90 and 12 (26.7%) at day 180 after treatment. |
| Corduff et al., 2023 ^28^ | Full-face and upper neck | **Elasticity**, **wrinkle**, **roughness**, **laxity, pore, ptosis, pigmentation**/ Scientific Assessment Scale of Skin Quality | Elasticity: Mean value of 2.28 at baseline and 1.74 post treatment (either 6 or 10 months).  Wrinkle: Mean value of 1.94 at baseline and 1.52 post treatment (either 6 or 10 months).  Roughness: Mean value of 2.1 at baseline and 1.83 post treatment (either 6 or 10 months).  Laxity: All patients experienced some improvement.  Pigmentation: Mean value of 1.83 at baseline and 1.7 post treatment (either 6 or 10 months).  Pore size: Mean value of 1.54 at baseline and 1.37 post treatment (either 6 or 10 months).  Ptosis: All patients experienced some improvement in skin and fibromuscular ptosis. |
| Tran et al., 2022 ^3^ | Lower face and neck | **Laxity**/ Facial Laxity Rating Scale | Facial Laxity: At baseline, 9 out of 10 patients (90.0%) had Moderate and one (10.0%) had Mild Facial Laxity in Lower Face and Neck. 3 months after treatment, 8 out of 9 patients (88.89%) had Mild Facial Laxity – Lower Face, and 1 patient (11.11%) had Moderate Facial Laxity – Lower Face. For the Neck area, 7 out of 9 patients (77.78%) had Mild Facial Laxity, whereas 2 (22.22%) had Moderate Facial Laxity. |
| ***Neck*** | | | |
| Jones et l., 2017 ^35^ | Neck | **Laxity-Investigators**/ The Neck Laxity Grading Scale (0 as None, No Loose Skin, Toned and Firm Skin with Smooth Skin Surface Texture, firmness to 4 as Extreme, Prominent redundancy of skin without underlying tone and severe wrinkling and crepiness on skin surface);  **Firmness-Patients**/ 11 point scale (0 as very firm to 10 as severe lack of firmness);  **Texture- Patients**/ 11 point scale (0 as no wrinkles or crepiness to 10 as severe wrinkling or crepiness);  **Laxity, Sagging Skin-Patients**/ 11 point scale (0 as no laxity or skin sagging to 10 as extreme laxity and skin sagging) | **MFU-V (Mean ± SD):**  Neck Laxity: 2.8±0.63 at baseline, 2.4±1.07 at day 30, 1.5±0.53 at day 90, and 1.4±0.7 at day 180 after treatment (Day 90 vs baseline; p<0.05 persistent to day 180).  Firmness, texture, and laxity/sagging: There was a decrease from baseline in the follow-up visits in these scores. The decrease became significant at day 30 for firmness, texture, and laxity/sagging skin (p = 0.0116, 0.0004, and 0.0049, respectively).  **MRF (Mean ± SD):**  Neck Laxity: 2.7±0.67 at baseline, 2.1±1.1 at day 30, 1.6±1.2 at day 90, and 0.86±0.7 at day 180 (Day 90 vs baseline; p<0.05 persistent to day 180).  Firmness, texture, and laxity/sagging: There was a decrease from baseline. This decrease became significant at day 180 for firmness (p = 0.0207), day 180 for texture (p = 0.0017), and day 90 for laxity/sagging skin (p = 0.0291).  There was no significant difference in mean self-assessed texture and laxity/sagging skin scores between the 2 treatments groups |
| Baumann et al., 2016 ^36^ | Submental, submandibular areas, lower neck | **Laxity-Investigators**/ Blinded comparison with baseline photographic images. 3-point scale.  **Wrinkle, sagging, tightening, smoothness, evenness-Patients**/ Questionnaire (Less Sagging, Smoother Skin Texture, More Even Skin Tone, Tighter/Lifted Under Chin.  **Neck Fold, neck sagging, texture, ptosis- Investigators)**/ L'Oreal Photographic Scale. | Laxity: At day 90 after treatment investigators reported 17 (53%) of patients as improved, 6 (9%) as no change; and 9 (28) improved, but incorrect pre- vs posttreatment image was selected.  Wrinkle, sagging, tightening, smoothness, evenness: At day 180, 68% of subjects reported continued improvement. Among the subjects, improvement in “Less Sagging on Cheeks/Jawline” and “Tighter/Lifted Under Chin” was noted at day 90 (52% for each category).  Horizontal Neck Folds (Mean ± SD): 3.0±1.0 at baseline, 2.6±1.1 at day 60; 2.6±1.2 at day 90 and 2.7±1.2 at day 180 after treatment.  Neck sagging (Mean ± SD): 3.5±0.8 at baseline, 2.9±1.0 at day 60; 2.9±1.1 at day 90 and 3.2±1.2 at day 180 after treatment.  Texture (Mean ± SD): 2.4±1.2 at baseline, 2.3±1.2 at day 60; 2.3±1.2 at day 90 and 2.3±1.2 at day 180 after treatment.  Ptosis (Mean ± SD): 3.2±1.0 at baseline, 2.7±1.1 at day 60; 2.8±0.9 at day 90 and 3.0±1.1 at day 180 after treatment. |
| ***Abdomen*** | | | |
| Lin et al., 2020 ^47^ | Lower abdomen | **Laxity- Patients and Investigators** / 5 point scale (0 no laxity to 4 very large amount laxity) | Laxity-Patients: Mean of 2 at baseline and 0.85 at 6 months after treatment.  Laxity-Investigators: Mean of 1.98 at baseline and 0.98 at 6 months after treatment. |
| ***Buttocks*** |  |  |  |
| Goldberg et al., 2014 ^43^ | Buttocks | **Sagging, wrinkles, smoothness, evenness-Patients**/ Questionnaire (Less sagging, Improvement in lines/wrinkles; Smoother skin texture; More even skin tone) | Noted improvements: 18 (66.7%) at day 90 and 14 (73.7%) at day 180 after treatment.  Less sagging: 16 (59.3%) at day 90 and 10 (52.6%) at day 180 after treatment.  Improvement in lines/wrinkles: 4 (14.8%) at day 90 and 5 (26.3%) at day 180 after treatment.  Smoother skin texture: 13 (48.1%) at day 90 and 6 (31.6%) at day 180 after treatment.  More even skin tone: 4 (14.8%) at day 90 and 3 (15.8%) at day 180 after treatment. |
| ***Arm*** | | | |
| Vachiramon et al., 2021 ^9^ | Upper Arm (posterior arm) ***_ Single plane*** | **Laxity-Investigator** / Investigator Assessment Skin Laxity Scoring System (0 as No loose skin, toned and firm skin with smooth skin surface texture to 4 as Prominent redundancy of skin without underlying tone, severe wrinkling, and crepiness on skin surface) | 24 patients were scored as level 2 and 3 as level 1 at baseline, 6 patients level 2, 18 level 1, 3 level 0 at month 1; 4 patients level 2, 19 level 1, 4 level 0 at month 3; and 19 patients level 2, 8 level 1 at month 6 after treatment. |
|  | Upper Arm (posterior arm) ***_ Dual plane*** | **Laxity-Investigator** / Investigator Assessment Skin Laxity Scoring System (0 as No loose skin, toned and firm skin with smooth skin surface texture to 4 as Prominent redundancy of skin without underlying tone, severe wrinkling, and crepiness on skin surface) | 24 patients were scored as level 2 and 3 as level 1 at baseline, 5 patients level 2, 21 level 1, 1 level 0 at month 1, 4 patients level 2, 19 level 1,4 level 0 at month 3; and 21 patients level 2, 8 level 1 at month 6 after treatment. |
| ***Elbow*** | | | |
| Rokhsar et al., 2015 ^42^ | Ellbow | **Tightening-Investigators**/ Blinded reviewers made qualitative comparisons of images of the treated elbows obtained at baseline and after treatment. If the reviewer perceived an improvement, they were asked to select the correct post-treatment photograph. | At day 90 after treatment, 9 (56%) patients were evaluated as improved and 4 (25%) as no change. Also, 3 (19%) patients were scored as improved but incorrect images were selected. |
| ***Décolletage*** | | | |
| Fabi et al., 2013 ^45^ | Décolletage | **Lines**, **sagging**, **tightening**, **texture**/ NM. Wrinkle/ Fabi/Bolton Chest Wrinkle Scale (FBCWS) | Lines/Wrinkles: 83% improvement at day 90 and 90% at day 180 after treatment.  Sagging: 50% improvement at day 90 and 29% at day 180 after treatment.  Improvement in Tightening/Lifting: 29% improvement at day 90 and 24% at day 180 after treatment.  Improvement in Texture; 25% improvement at day 90 and 33% at day 180 after treatment.  (day 90 vs baseline; p<0.0001).  (day 180 vs baseline; p=0.003).  All 24 participating subjects had a FBCWS score of 3 or higher at day 0. At day 90, 11 of 24 subjects (46%) had a FBCWS score of 1 or 2; by day 180, 13 of 21 subjects (62%) had a score of 1 or 2 (P<0.0001 for trend of decreasing FBCWS score over time). |
| Fabi et al., 2015 ^46^ | Décolletage | **Wrinkle-Investigators**/ Changes were defined as improvements that were (1) striking, substantial, and immediately noticeable, (2) readily apparent but modest in nature, (3) slight and subtle in nature and may require close examination, or (4) no change. If an improvement was noted, the pre-treatment and post-treatment photographs were identified by the clinician. | Based on blinded assessments, 79 subjects (69.9%) demonstrated aesthetic improvement at day 90 post-treatment, and 72 (66.7%) did so at the 180-day time point. Two subjects achieving substantial improvement. Among the remaining subjects, 26 (22.4%) showed no difference between baseline and follow-up visits. For 13 subjects (11.2%), the blinded reviewers selected incorrect post-treatment photographs. |
| Fabi et al.,. 2020 ^44^ | Décolletage | **Wrinkle** (dynamic and at rest)/ validated Merz Décolletage Wrinkle Scales | The Merz Décolletage Wrinkle-Dynamic score (mean ± SD): Decreased from 2.45±0.37 at baseline to 1.89±0.22 at week 12, 1.93±0.21 at day 180, and 1.88±0.12 at day 360. These changes represent a significant 23.3% decrease at week 12 (P≤0.01), which remained significant at day 180 (P<0.01) and day 360 (P<0.01).  The Merz Décolletage Wrinkle–At Rest score (mean ± SD): Decreased from 2.0±0.11 at baseline to 1.56±0.26 at week 12, 1.4±0.83 at day 180, and 1.38±0.38 at day 360. These changes represent a significant 22.5% decrease from baseline at week 12 (P≤0.01) and these differences remained significant at day 180 (P=0.01) and day 360 (P=0.01) |
| ***Knee*** | | | |
| Gold et al., 2014 ^48^ | Above the knees | **Smoothness**, **evenness**, **sagging-Patients**/ Questionnaire (Improvement noticed, notice improvement in lines and wrinkles, less sagging, smoother skin texture, more even skin tone)  **Tightening-Patients**/GAIS | Smoothness, evenness, sagging:  Improvement noticed: 24 (85.7%) patients responded yes and 4 (14.2%) no at day 90 and 19 (67.8%), No: 9 (28.6%) at day 180 after treatment.  Lines and wrinkles: 14 (50.0%) patients noticed improvement at day 90 and 14 (50.0%) at day 180 after treatment.  Sagging skin: 11 (39.3%) patients reported less sagging at day 90 and 11 (39.3%) at day 180 after treatment.  Skin texture: 14 (50.0%) patients reported smoother skin at day 90 and 6 (21.4%) at day 180 after treatment.  Even skin tone: 2 (7.1%) patients reported more even skin at day 90 and 4 (14.3%) at day 180 after treatment.  Tightening: 24 subjects (86%) showed improved tightening of knee skin laxity at each of the days 90 and 180. 3 subjects showed no change and the appearance of one subject worsened. |
| ***Combination of several body parts*** | | | |
| Chang et al., 2019 ^40^ | Full face, Full face/neck, Lower face, Lower face/neck, Jawline, Brow, Neck, Chest, Body | **Tightening- Patients**/ None, mild [0–25%], moderate [26–50%], significant [51–75%], dramatic [76–100%] | 17 (20.5%) patients scored the scale as none; 31 (37.3%) and mild; 23 (27.7%) as moderate; 12 (14.5%) as significant; and 0 (0.0%) as dramatic. |
| Sasaki et al., 2012 ^37^ | Forehead, Periorbitum, Face, Neck, Décolleté | **Wrinkle-Investigators**/ Class I mild, score 1 - 3; Class II moderate 4 - 6. | Periorbitum: 9 patients as Class I and 10 patients as Class II 6 months after treatment.  Décolletage: 2 patients as Class I and 3 Patients as Class II 6 months after treatment.  Brachium: 15 patients as Class I and 29 as Class II) 6 months after treatment.  Periumbilicus: 2 patients as Class I and 4 as Class II) 6 months after treatment.  Inner Thigh : 1 patient as Class II 6 months after treatment.  Knees: 2 patients as Class I and 2 as Class II 6 months after treatment.  Hand: 1 patient as Class II 6 months after treatment.  Buttock: 2 patient as Class II 6 months after treatment. |
| Casabona et al., 2019 ^39^ |  | **Manchester Scar Scale scores (components: color, contour, distortion, texture, and finish)/** Manchester Scar Scale scores. All components were scored on a scale of 1 to 4, except for finish, which was scored either 1 (matte) or 2 (shiny). These scores range from a possible high of 18 points to a low of five points, with lower scores indicating better aesthetic appearance. | The mean ± SD pretreatment Manchester Scar Scale score decreased from 9.35±1.18 to 6.30±1.26 after 90 days (p<0.001). |

MFU-V: Microfocus ultrasound with visualization; MRF: monopolar capacitive-coupled radiofrequency; SD: Standard deviation.

**Supplemental Table 10.** Summary of adverse events following MFU-V treatment.

| **Author, year** | **Summary of finings** |
| --- | --- |
| ***Facial regions*** | |
| Lim et al., 2023 ^18^ | No permanent or serious side effects were noted during the study. No patient experienced post-inflammatory hyperpigmentation or worsening of melasma. |
| Maas et al., 2019 ^19^ | No evidence of skin damage or other sequelae such as scarring, burns, or hypo- or hyper-pigmentation. |
| Jeon et al., 2020 ^20^ | Approximately half of the patients reported swelling, which were mild in severity. Other adverse events observed were bruising (reported by 5 patients), nodules (reported by 2 patients), ectropion (reported by 1 patient), and unilateral dacryorrhea (reported by 1 patient). All adverse effects were mild and resolved within 2 weeks. No serious adverse events were reported. |
| Wanitphakdeedecha et al., 2020 ^21^ | All patients developed mild erythema immediately after the treatment with spontaneously resolved at 1-week follow-up. No post-inflammatory hypo- or hyperpigmentation, bullous formation, scar, crusting, oozing, and any serious adverse events were recorded in this study. |
| Oni et l., 2014 ^22^ | Throughout the study, there were no reports of acute skin damage or long-term sequelae such as scarring, burns, hypopigmentation, hyperpigmentation, or ulceration. Seven adverse events were reported during the study. 3 device-related adverse events were wheals on the cheek or neck, all of which were rated as mild and resolved spontaneously with no sequelae. The duration of facial swelling did not exceed 7 to 10 days in any patient, and there were no reports of crusting, pigment changes, or persistent pain. |
| Lee et al,. 2015 ^23^ | Thirty-five patients immediately presented with slight erythema and edema after treatment, and three patients immediately presented with moderate erythema and edema after treatment. In all affected patients, both erythema and edema completely resolved by 2 days after treatment. Two patients presented with red linear striations of the check after treatment with the 3 mm transducer. They were treated using focal cooling without sequelae such as pigmentation and textural abnormalities. Hypopigmentation, hyperpigmentation, ulceration, and erosion were not present in any patients. There were no adverse events, such as nerve or muscle dysfunction, severe pain, bruising, and bleeding. |
| Yalici-Armagan et al., 2020 ^24^ | One subject reported transient stinging sensation/dysesthesia on the face after the procedure that lasted approximately 6 months. Another subject reported erythema and striation after application. No serious adverse events were observed. No subject had a severe adverse effect such as nerve and muscle dysfunction in the current study. Although anesthetics or analgesics were not used in our study, subjects experienced only minimal pain during the treatment. |
| Araco et al., 2020 ^25^ | No major side effects were reported during the study. |
| Vachiramon et al., 2021 ^13^ | No major side effects were reported during the study. |
| Schlessinger et al., 2019 ^8^ | Notable side effects include bruising (44%), tenderness/ soreness (43%), and redness (35%) Other events included edema/welts/swelling (9.3%), paresthesia/numbness (5.2%). Adverse events severity was rated as mild (n=167) and moderate (n=4).  Subjects that received two treatments reported more adverse events than those with one treatment. |
| ***Facial and Neck regions*** | |
| Fabi et al., 2014 ^26^ | There was no evidence of acute skin damage or long-term sequelae such as scars, burns, or hypo- or hyperpigmentation. There were no reports of serious or permanent adverse events. |
| Shome et al., 2019 ^27^ | Almost all the patients had swelling that persisted for 2 to 14 days. No other adverse events, including but not limited to nerve and muscle dysfunction, facial fat deformity, scarring, or bleeding were observed. |
| Corduff et al., 2023 ^28^ | Five patients developed superficial welts after the first superficial treatment, which resolved within a few days of initiating antihistamine therapy. Three patients experienced erythema lasting a few days, three reported immediate posttreatment tenderness, and two experienced mild bruising not requiring treatment. No severe or permanent adverse events were reported.  The mean erythema level was 1.89 before treatment and 1.87 after treatment. |
| Harris et al., 2015 ^29^ | One was described as prolonged erythema with mild scabbing and determined to be of moderate severity. The other 2 events were considered mild and consisted of raised areas of mild edema or welts. All 3 adverse events were considered to be owing to localized, suboptimal coupling and/or contact with the skin during treatment. All 3 events resolved after 90 days without sequelae. No cases of acute skin damage or long-term sequelae, such as scarring, burns, hypopigmentation, hyperpigmentation, or ulceration, were reported during the study. There were no neurologic adverse events. |
| Kerscher et al., 2019 ^30^ | No unexpected event were reported, and treatment were well tolerated. Slight erythema was seen in fairer skin phototypes immediately after treatment, which lasted for approximately 30 to 60 min. No serious adverse events were reported. |
| Kerscher et al., 2019 ^31^ | No adverse events occurred during the course of the evaluation, and no patients withdrew from the evaluation due to an adverse event. Dermal edema in the treatment area was observed 3 days after treatment but resolved completely by week 4. Sonography illustrated a representative example of the course of edema.  The mean erythema level (Mexameter Units) at baseline was 313.70±76.11, and was increased slightly immediately after treatment to 326.45±72.84 and decreased at day 3 after treatment to were further decreased at week 4 (293.63±69.51 MU) and at week 12 (290.31±62.34 MU). Erythema values did not demonstrate significant changes compared to baseline for short-term or long-term evaluation. |
| Werschler et al., 2016 ^32^ | Among the eight reported adverse events, only swelling under right eye (n=1) was considered to be possibly treatment-related and it resolved within four days. There were no serious adverse events. |
| Lu et al., 2017 ^33^ | The most common adverse event was bruising over the bony prominence area which was observed in 80% of population. The one case of burn occurred due to improper application of the ultrasound probe. None of the cases reported paresthesia, numbness, or tingling after the treatment. |
| Alhaddad et al., 2019 ^34^ | One patient was noted to have Grade 1 erythema (barely visible and localized) at day 30. The erythema resolved before day 90 visit. No patients were noted to develop bruising, edema, or contour irregularity at day 30 through day 180 visit. |
| Fusano et al., 2021 ^11^ | No significant difference was noticed in terms of post procedure erythema and edema, while no adverse events occurred. |
| Tran et al., 2022 ^3^ | Side effects consisted of bruising (40%; n=4), increased warmth at treatment site (20%; n=2), tenderness (20%; n=2), edema (10%; n=1), intermittent facial muscle numbness (10%; n=1), and dizziness during treatment (10%; n=1), all were mild in nature, limited to the treatment area, and improved without intervention. No serious adverse events were reported. |
| Polacco et al., 2020 ^5^ | There were no adverse events such as persistent erythema, bruising, welting, or skin ulcerations. |
| Palm et al., 2018 ^7^ | There were no adverse events reported by any of the patients. |
| ***Neck*** | |
| Jones et al., 2017 ^35^ | **MFU-V:**  Four of 10 (40%) patients were noted to have edema at day 30, with a mean severity score in these patients of 2 (range 1–3). One patient (10%) experienced Grade 2 erythema at day 30. Two patients (20%) developed contour irregularity, where one was recorded as Grade 3 at day 30 and the other Grade 1 at day 90. All adverse events in patients resolved by day 180. No patients were noted to develop bruising.  **MRF:**  One patient was noted to have Grade 1 edema (barely visible and localized) at day 30. This patient did not follow up at days 90 and 180. No patients were noted to develop bruising.  **MFU-V vs MRF:**  Although more MFU-V–treated patients experienced edema, there was no statistically significant difference in edema at day 30 between the 2 treatment groups (p = 0.098). |
| Baumann et al., 2016 ^36^ | One subject described numbness in lower neck on the day treatment that resolved after 5 days; the same subject then reported throbbing pain in lower neck that persisted for 3 weeks. Both events were considered mild in severity. |
| Vachiramon et al., 2020 ^6^ | No complications such as post inflammatory hyperpigmentation regarding the anesthetic application or procedure were noted |
| ***Combination of several body parts*** | |
| Sasaki et al., 2012 ^37^ | All patients developed fleeting erythema immediately after treatment, especially around the upper and lower eyelids. All erythema dissipated within a few hours. Transient edema from therapy was difficult to assess because of subcutaneous injections of local anesthetic solution for pain management. No other adverse events (such as blistering, ulceration, scarring, dyschromia, numbness, bruising, or striations) were noted at any time point. |
| Montes et al., 2019 ^38^ | On a scale of 1 to 10, 75% of patients believed the treatment process was excellent (score of 10). Treatment was very well-tolerated as most patients (69%) reported no treatment-related adverse events. The adverse events that did occur consisted of local events that are commonly observed after treatment. |
| Casabona et al., 2019 ^39^ | There were no reported adverse events. |
| Chang et al., 2019 ^40^ | Most common side effects reported were tenderness (49%), swelling (32%), redness (27%), and bruising (17%). Of note, 27% of responders reported no post procedure side effects. |
| Sasaki e al., 2017 ^41^ | Transient erythema was observed for 1 to 2 hours along with mild swelling for several days in all treated areas. |
| ***Elbow*** | |
| Rokhsar et al., 2015 ^42^ | There was no evidence of acute skin damage or any long-term sequelae such as scarring, burns, ulceration, or hypopigmentation or hyperpigmentation. |
| ***Buttocks*** | |
| Goldberg et al., 2014 ^43^ | Three adverse events were reported by 3 subjects. One report of lightheadedness. The other event was a burn to the buttock that resulted in scarring, which was believed to be due to poor contact between the skin and transducer. There was no evidence of erythema or edema in any other subjects. Reports of significant subject discomfort were a common finding. |
| ***Arm*** | |
| Vachiramon et al., 2021 ^9^ | Adverse events were limited to erythema and edema in all patients. Erythema subsided within 1 day while edema lasted for 1–3 days in all patients. |
|  |  |
| Fabi et al., 2013 ^45^ | Erythema and edema were acute responses and were mild to moderate and transient. Only 1 non serious adverse event was noted, where a subject immediately developed indurated, erythematous linear plaques, corresponding to sites of MFU-V line delivery with the 7.0-MHz, 3.0 mm depth transducer. The plaques resolved in 3 weeks with no sequelae. |
| Fabi et al., 2015 ^46^ | The most common adverse events were mild and transient tenderness (47.4%), bruising (23.2%), pruritus (16.8%), and edema (4.2%). All were transient in duration and mild in severity, except for 2 events of moderate severity however, only 1 (bruising) was treatment related. The 1 moderate bruising resolved in 20 days. There was no evidence of any acute effects on the treated area such as edema or erythema and no long-term effects such as hypo- pigmentation or hyperpigmentation. |
| ***Abdomen*** | |
| Lin et al., 20202 ^47^ | One patient reported skin welting, which fully resolved after 2 weeks requiring no further intervention. No significant adverse events were recorded. |
| Vachiramon et al., 2020 ^10^ | Erythema and edema were noted in all treated sites, which resolved spontaneously within 1–2 weeks. Bruising was observed along local anesthesia injection sites, which also resolved spontaneously within 10 days. Skin tenderness was observed by all patients on both treatment sides, which spontaneously subsided in 2 weeks without any treatment. No other adverse events were found. |
| ***Knee*** | |
| Gold et al., 2014 ^48^ | No evidence of skin injury was noted by the investigator. The investigator did not observe any sign of erythema, edema, or bruising following treatment and there were no subject reports of adverse events. |

**Supplemental Table 11.** Summary of eyebrow height following MFU-V treatment.

| **Author, year** | **Treated region** | **Reported outcomes/ Assessment methods** | **Summary of findings** |
| --- | --- | --- | --- |
| Wanitphakdeedecha et al., 2020 ^21^ | Upper face | **Eyebrow height**/ ImageJ Software | The average eyebrow height elevation was 1.51 mm at 1-month, 1.25 at 3-month, and 1.22 mm at 6-month follow-up (vs baseline, all p<0.001). |
| Lu et al., 2017 ^33^ | Face and neck | **Eyebrow height/** calculated as the average vertical distance of medial canthus, medial limbus, lateral limbus, and lateral canthus to the highest point of brow. | Mean value of 29.07 mm at baseline, 29.54 mm at day 90, 28.95 mm at day 180 after treatment (vs baseline, p=0.02, p=0.65) |
| Sasaki et al., 2012 ^37^ | Forehead, Periorbitum, Face, Neck, Décolleté | **Eyebrow lift**/ Matched Orientation Function of  Mirror Software | An average elevation between 1 - 2 mm (7% - 8% increase from baseline) in each patients at month 6 after treatment. |
| Corduff et al., 2023 ^28^ | Full-face and upper neck | **Eyebrow height** / A line was drawn to connect the two inner canthi. Points were drawn at the upper margin of the brow at the medial, lateral, and central points and two points were marked halfway. Digital guides were marked at intercanthal line intersections. The distance was measured between the eyebrow height and the corresponding point on the intercanthal line | Among 10 patients 8 experienced an average of 1.7 mm increase in mean brow height and 1.8 mm in maximum brow height (either 6 or 10 months after treatment). |

MFU-V: Microfocus ultrasound with visualization.

**Supplemental Table 12.** Summary of other reported findings following MFU-V treatment.

| **Author, year** | **Treated region** | **Reported outcomes/ Assessment methods** | **Summary of finings** |
| --- | --- | --- | --- |
| ***Facial regions*** | | | |
| Wanitphakdeedecha et al., 20202 ^21^ | Upper face | **Facial volume**/ Vectra H1 Imaging System | Difference of volume compared to baseline (Mean ± SD): 0.15±0.77 at week 1, 0.57±0.76 at month 1, 0.45±0.59 at month 3, and 0.36±0.63 at month 6 after treatment (vs baseline; p=0.18, p=0.96, p=0.372) |
| Lim et al., 2020 ^18^ | Both cheeks | **Lightening of melasma**/ 6-Point scale (No change, 0-25%lighter, 26-50% lighter, 51-75%lighter).  **Area of melasma**/ Grading the area of melasma involvement per treatment site, as: <10%, 10%–­29%, 30%–49%, 50%–­69%, 70%–­89% and 90%–­100% involvement.  **Melasma Area and Severity Index (mMASI)/** mMASI was calculated for each treatment site by visually assessing the area of involvement and darkness of melasma, where mMASI = area of involvement x darkness | Lightening of melasma: 40% of sites improved at month 1; specific percentages for 1%-25% and >50% lightening not provided. 72.5% of sites improved at month 4; most had 1%-25% lightening, 2.5% had >50% lightening. At month 5, specific percentages not provided; lightening maintained except for one site with recurrence.  Area of melasma: At baseline, the area of melasma involvement was less than 10% in 15% of cheeks and 10%–­89% in the rest, including 42.5% of sites with melasma occupying 30%–­49% of the cheek, and 2.5% of sites with melasma covering 70%–­89% of the cheek. At study closure, improvements were seen in area of melasma involvement –­ melasma covering 50%–­69% of cheeks declined from 10% to 2.5% of sites, while melasma covering less than 10% of the cheek increased from 15% to 27.5% of sites, and melasma covering 10%–29% of the cheek increased from 30% to 52.5% of sites. Reduction in melasma surface area occurred in 40% of sites 1 month after the second treatment, and in 70% of sites by month 5. No increase in area of melasma involvement occurred.  mMASI: The baseline mean was 13.2 ± 5.26. Mean mMASI at each follow-up demonstrated a clear downward trend from baseline, with maximum improvement noted at the month 4 (mean mMASI was 2.4 ± 2.2). A minor increase in mean mMASI occurred at month 5, 2.8 ± 2.24 but was still significantly lower compared to baseline (vs baseline; p < 0.0001 at all time points). |
| Jeon et al., 2020 ^20^ | Lower eyelid | **Bulging severity score**/ grade 0, no improvement; grade 1, <20%; grade 2, 20%-39%; grade 3, 40%-59%; grade 4, 60%-79%; and grade 5, 80%-100%. Also treatment response grades were collapsed into a single ordinal variable by summing the number of individual grades by 3 dermatologists in the minimal (0-2), fair (3-5), and good (6 or more) responses | The median bulging severity score was 9.0 (IQR 7.0-10.0), and median treatment response score was 4.0 (IQR 3.0-5.0). Overall, when evaluated by photographs taken with the conventional digital camera, 25 (13.1%) have had a minimal response, 119 (62.3%) a fair response, and 47 (24.6%) a good response. The proportion of patients with good response tended to increase with the number of treatments. |
| Maas et al., 2019 ^19^ | Cheeks and/or temples | **Appearance of scars/** compared to images obtained at baseline, outcome was determined by a blinded, qualitative comparison of facial images obtained after the MFU-V treatment. 5 -point scale (exacerbation (-1), to 76 to 99% improvement (4)). If improvement was assessed, the reviewer was asked choose the correct post-treatment image.  **Severity of Acne scar- Patients** and **Investigators /** Acne Scar Improvement Scale Score, 6-point scale (1% to 25% improvement, 26% to 50% improvement, 51% to 75% improvement, 76% to 99% improvement Acne scar exacerbation, No change in appearance) | Appearance of scars: Among the 90-day images available for blinded assessments (n=11), all were determined to be improved (100%), and the post-treatment image was correctly selected for 64%. Among the available 180-day images (n=15), all were assessed as improved, and a correct post-treatment image was selected for 40%.  Severity of Acne scar-Patients: All subjects noted some improvement in acne-scar severity at the 60-day assessment, and one subject each indicated no change at the 90- and 180-day assessments. All subjects (100%) noted some degree of improvement at the 60-day assessment, decreasing to 83% and 89% at the 90- and 180-day assessments, respectively.  Severity of Acne scar-Investigators: Most subjects were determined to have 25 to 50% improvement. One subject had 75 to 99% improvement at day 180. No subjects had exacerbation in their acne scars, and none showed any change in their skin appearance. |
| ***Facial and Neck regions*** | | | |
| Corduff et al., 2023 ^28^ | Full-face and upper neck | **Submental lift**/ calculated using a lateral image and an enclosed area between the lower neckline above the thyroid notch to the point where the chin joined the neck | The mean submental lift per patient was 78.7 mm2 |
| ***Arm*** | | | |
| Vachiramon et al., 2021 ^9^ | Upper Arm (posterior arm) ***_ Single plane*** | **Arm circumferences**/ arm circumferences were also measured at the midpoint between the shoulder (acromial angle) and the elbow (olecranon) using a single standard measuring tape. | A modest reduction of arm circumferences was observed. For single‐plane treatment, the mean arm circumferences were 28.37 (±2.76) cm, 27.83 (±2.63) cm, 27.77 (±2.49) cm, and 27.79 (±2.48) cm for baseline, 1, 3, and 6 months. |
|  | Upper Arm (posterior arm) ***_ Dual plane*** | **Arm circumferences**/ arm circumferences were also measured at the midpoint between the shoulder (acromial angle) and the elbow (olecranon) using a single standard measuring tape. | A modest reduction of arm circumferences was observed. For dual‐plane treatment, the mean arm circumferences were 28.26 (±2.47) cm, 27.72 (±2.55) cm, 27.71 (±2.48) cm, and 27.90 (±2.40) cm at 6 months |

MFU-V: Microfocus ultrasound with visualization; SD: Standard deviation.

**REFRENCES:**

1. Sterne JAC, Savović J, Page MJ, et al. RoB 2: a revised tool for assessing risk of bias in randomised trials. *Bmj*. Aug 28 2019;366:l4898.

2. Sterne JA, Hernán MA, Reeves BC, et al. ROBINS-I: a tool for assessing risk of bias in non-randomised studies of interventions. *Bmj*. Oct 12 2016;355:i4919.

3. Tran J, Lultschik S, Sapra S, Dong K. Prospective, Single-arm, Split-face Pain Management Evaluation of Nitrous Oxide System During Micro-Focused Ultrasound With Visualization. Randomized Controlled Trial. *J Drugs Dermatol*. Nov 01 2022;21(11):1228-1234. doi:<https://dx.doi.org/10.36849/JDD.7030>

4. Vachiramon V, Palakornkitti P, Anuntrangsee T, Rutnin S, Visessiri Y, Fabi S. A comparative study of pain perception during the microfocused ultrasound procedure between topical anesthesia and combined topical anesthesia with forced air cooling. Randomized Controlled Trial. *J*. Apr 2023;22(4):1279-1285. doi:<https://dx.doi.org/10.1111/jocd.15568>

5. Polacco MA, Butz DR, Bass R, Luu T, Kurum E, DiCarlo A, Maas CS. Nerve Blocks Prior to Microfocused Ultrasound Treatment are Safe and Reduce Patient Discomfort. *Aesthet*. 07 13 2020;40(8):887-891. doi:<https://dx.doi.org/10.1093/asj/sjaa031>

6. Vachiramon V, Tanratana P, Anuntrangsee T, Palakornkitti P, Yeesibsean N, Kungvalpivat P, Fabi S. The role of topical capsaicin gel in pain management during microfocused ultrasound treatment for neck laxity. *Skin Res Technol*. Jan 2023;29(1):e13240. doi:<https://dx.doi.org/10.1111/srt.13240>

7. Palm MD, Misell LM. Topical Transdermally Delivered Lidocaine and Benzocaine Compared to Compounded Lidocaine/Tetracaine During Microfocused Ultrasound With Visualization Treatment. Comparative Study

Equivalence Trial

Randomized Controlled Trial. *J Drugs Dermatol*. Jul 01 2018;17(7):729-734.

8. Schlessinger J, Lupin M, McDaniel D, George R. Safety and Effectiveness of Microfocused Ultrasound for Treating Erythematotelangiectatic Rosacea. Randomized Controlled Trial. *J Drugs Dermatol*. Jun 01 2019;18(6):522.

9. Vachiramon V, Triyangkulsri K, Iamsumang W, Chayavichitsilp P. Single-Plane Versus Dual-Plane Microfocused Ultrasound With Visualization in the Treatment of Upper Arm Skin Laxity: A Randomized, Single-Blinded, Controlled Trial. Randomized Controlled Trial. *Lasers Surg Med*. 04 2021;53(4):476-481. doi:<https://dx.doi.org/10.1002/lsm.23307>

10. Vachiramon V, Triyangkulsri K, Iamsumang W, Chayavichitsilp P. Efficacy and Safety of Microfocused Ultrasound With Visualization in Abdominal Skin Laxity: A Randomized, Comparative Study. Case Reports

Randomized Controlled Trial. *Lasers Surg Med*. 11 2020;52(9):831-836. doi:<https://dx.doi.org/10.1002/lsm.23234>

11. Fusano M, Galimberti MG, Bencini M, Fusano I, Bencini PL. Comparison of microfocused ultrasound with visualization for skin laxity among vegan and omnivore patients. *J*. Sep 2021;20(9):2769-2774. doi:<https://dx.doi.org/10.1111/jocd.13961>

12. Yusova ZY, Stepanova TV, Baranova EL, Demidion DV. Correction of involutional skin changes using microfocused ultrasound combined with PRP-therapy. Article. *Electro J Gen Med*. 2019;16(6)doi:10.29333/ejgm/115851

13. Vachiramon V, Namasondhi A, Anuntrangsee T, Kositkuljorn C, Jurairattanaporn N. A study of combined microfocused ultrasound and hyaluronic acid dermal filler in the treatment of enlarged facial pores in Asians. Randomized Controlled Trial. *J*. Nov 2021;20(11):3467-3474. doi:<https://dx.doi.org/10.1111/jocd.14360>

14. Higgins JP, Thomas J, Chandler J, Cumpston M, Li T, Page MJ, Welch VA. *Cochrane handbook for systematic reviews of interventions*. John Wiley & Sons; 2019.

15. Begg CB, Mazumdar M. Operating characteristics of a rank correlation test for publication bias. *Biometrics*. 1994:1088-1101.

16. Egger M, Smith GD, Schneider M, Minder C. Bias in meta-analysis detected by a simple, graphical test. *Bmj*. 1997;315(7109):629-634.

17. Higgins JPT, Thompson SG, Deeks JJ, Altman DG. Measuring inconsistency in meta-analyses. *Bmj*. 2003;327(7414):557-560.

18. Lim JTE. Safety and efficacy of superficial micro-focused ultrasound with visualization for melasma in Asians: An uncontrolled pilot study. *J*. Jun 2023;22(6):1764-1773. doi:<https://dx.doi.org/10.1111/jocd.15661>

19. Maas CS, Joseph JH. Safety and Effectiveness of Microfocused Ultrasound With Visualization for the Correction of Moderate to Severe Atrophic Acne Scars. Clinical Trial. *J Drugs Dermatol*. Nov 01 2019;18(11):1109-1114.

20. Jeon HC, Kim DY, Jin SP, Lee DH. A new treatment protocol of microfocused ultrasound for lower eyelid fat bulging. *J Dermatolog Treat*. Dec 2021;32(8):1005-1009. doi:<https://dx.doi.org/10.1080/09546634.2020.1720581>

21. Wanitphakdeedecha R, Yan C, Ng JNC, et al. The efficacy of macro-focused ultrasound in the treatment of upper facial laxity: A pilot study. Article. *J Cosmet Dermatol*. Aug 2020;19(8):1955-1961.

22. Oni G, Hoxworth R, Teotia S, Brown S, Kenkel JM. Evaluation of a microfocused ultrasound system for improving skin laxity and tightening in the lower face. Clinical Trial

Research Support, Non-U.S. Gov't. *Aesthet*. Sep 2014;34(7):1099-110. doi:<https://dx.doi.org/10.1177/1090820X14541956>

23. Lee I, Nam S, Park E, Kim Y. *Evaluation of micro-focused ultrasound for lifting and tightening the face*. Archives of Aesthetic Plastic Surgery. e-aaps.org; 2015.

24. Yalici-Armagan B, Elcin G. Evaluation of microfocused ultrasound for improving skin laxity in the lower face: A retrospective study. *Dermatol Ther*. 11 2020;33(6):e14132. doi:<https://dx.doi.org/10.1111/dth.14132>

25. Araco A. Prospective Study on Clinical Efficacy and Safety of a Single Session of Microfocused Ultrasound With Visualization for Collagen Regeneration. *Aesthet*. 09 14 2020;40(10):1124-1132. doi:<https://dx.doi.org/10.1093/asj/sjz363>

26. Fabi SG, Goldman MP. Retrospective evaluation of micro-focused ultrasound for lifting and tightening the face and neck. Research Support, Non-U.S. Gov't. *Dermatol Surg*. May 2014;40(5):569-75. doi:<https://dx.doi.org/10.1111/dsu.12471>

27. Shome D, Vadera S, Ram MS, Khare S, Kapoor R. Use of Micro-focused Ultrasound for Skin Tightening of Mid and Lower Face. *Plast*. Dec 2019;7(12):e2498. doi:<https://dx.doi.org/10.1097/GOX.0000000000002498>

28. Corduff N, Lowe S. Hi5 Protocol for the Use of Microfocused Ultrasound with Visualization. *Plast reconstr surg, Glob open*. Aug 2023;11(8):e5184. doi:10.1097/gox.0000000000005184

29. Harris MO, Sundaram HA. Safety of Microfocused Ultrasound With Visualization in Patients With Fitzpatrick Skin Phototypes III to VI. Research Support, Non-U.S. Gov't. *JAMA Facial Plast Surg*. Sep-Oct 2015;17(5):355-7. doi:<https://dx.doi.org/10.1001/jamafacial.2015.0990>

30. Kerscher M, Nurrisyanti AT, Eiben-Nielson C, Hartmann S, Lambert-Baumann J. Clinical and Biophysical Outcomes of Combining Microfocused Ultrasound with Visualization and Calcium Hydroxylapatite Filler for Facial Treatment. *Dermatol Ther (Heidelb)*. Mar 2019;9(1):135-142. doi:<https://dx.doi.org/10.1007/s13555-018-0273-y>

31. Kerscher M, Nurrisyanti AT, Eiben-Nielson C, Hartmann S, Lambert-Baumann J. Skin physiology and safety of microfocused ultrasound with visualization for improving skin laxity. *Clin Cosmet Investig Dermatol*. 2019;12:71-79. doi:<https://dx.doi.org/10.2147/CCID.S188586>

32. Werschler WP, Werschler PS. Long-term Efficacy of Micro-focused Ultrasound with Visualization for Lifting and Tightening Lax Facial and Neck Skin Using a Customized Vectoring Treatment Method. *J Clin Aesthet Dermatol*. Feb 2016;9(2):27-33.

33. Lu PH, Yang CH, Chang YC. Quantitative Analysis of Face and Neck Skin Tightening by Microfocused Ultrasound With Visualization in Asians. *Dermatol Surg*. Nov 2017;43(11):1332-1338. doi:<https://dx.doi.org/10.1097/DSS.0000000000001181>

34. Alhaddad M, Wu DC, Bolton J, Wilson MJ, Jones IT, Boen M, Goldman MP. A Randomized, Split-Face, Evaluator-Blind Clinical Trial Comparing Monopolar Radiofrequency Versus Microfocused Ultrasound With Visualization for Lifting and Tightening of the Face and Upper Neck. Randomized Controlled Trial. *Dermatol Surg*. 01 2019;45(1):131-139. doi:<https://dx.doi.org/10.1097/DSS.0000000000001653>

35. Jones IT, Guiha I, Goldman MP, Wu DC. A Randomized Evaluator-Blinded Trial Comparing Subsurface Monopolar Radiofrequency With Microfocused Ultrasound for Lifting and Tightening of the Neck. Comparative Study

Randomized Controlled Trial. *Dermatol Surg*. Dec 2017;43(12):1441-1447. doi:<https://dx.doi.org/10.1097/DSS.0000000000001216>

36. Baumann L, Zelickson B. Evaluation of Micro-Focused Ultrasound for Lifting and Tightening Neck Laxity. Evaluation Study. *J Drugs Dermatol*. May 01 2016;15(5):607-14.

37. Sasaki G, Tevez A. *Microfocused ultrasound for nonablative skin and subdermal tightening to the periorbitum and body sites: preliminary report on eighty-two patients*. scirp.org; 2012.

38. Montes JR, Santos E. Patient Satisfaction Following Treatment With Microfocused Ultrasound With Visualization: Results of a Retrospective Cross-Sectional Survey. *J Drugs Dermatol*. 01 01 2019;18(1):75-79.

39. Casabona G. Microfocused Ultrasound with Visualization for the Treatment of Stretch Marks. *J Clin Aesthet Dermatol*. Feb 2019;12(2):20-24.

40. Chang YC, Croix J, Javvaji S, Hernandez S, Chapas AM, MacGregor J. Patient satisfaction and our clinical experience with 459 microfocused ultrasound treatments. *Lasers Surg Med*. 08 2019;51(6):495-499. doi:<https://dx.doi.org/10.1002/lsm.23058>

41. Sasaki GH, Abelev N, Papadopoulos L. A Split Face Study to Determine the Significance of Adding Increased Energy and Treatment Levels at the Marionette Folds. *Aesthet*. Sep 01 2017;37(8):947-960. doi:<https://dx.doi.org/10.1093/asj/sjx055>

42. Rokhsar C, Schnebelen W, West A, Hornfeldt C. Safety and Efficacy of Microfocused Ultrasound in Tightening of Lax Elbow Skin. Research Support, Non-U.S. Gov't. *Dermatol Surg*. Jul 2015;41(7):821-6. doi:<https://dx.doi.org/10.1097/DSS.0000000000000390>

43. Goldberg DJ, Hornfeldt CS. Safety and efficacy of microfocused ultrasound to lift, tighten, and smooth the buttocks. Research Support, Non-U.S. Gov't. *Dermatol Surg*. Oct 2014;40(10):1113-7. doi:<https://dx.doi.org/10.1097/DSS.0000000000000126>

44. Fabi SG, Boen M, Alhaddad M, Goldman MP. Clinical Trial Evaluating the Long-Term Efficacy of Microfocused Ultrasound With Visualization for Decollete Rejuvenation. Clinical Trial. *J Drugs Dermatol*. Nov 01 2020;19(11):1026-1029. doi:<https://dx.doi.org/10.36849/JDD.2020.5265>

45. Fabi SG, Massaki A, Eimpunth S, Pogoda J, Goldman MP. Evaluation of microfocused ultrasound with visualization for lifting, tightening, and wrinkle reduction of the decolletage. Evaluation Study

Research Support, Non-U.S. Gov't. *J Am Acad Dermatol*. Dec 2013;69(6):965-71. doi:<https://dx.doi.org/10.1016/j.jaad.2013.06.045>

46. Fabi SG, Goldman MP, Dayan SH, Gold MH, Kilmer SL, Hornfeldt CS. A prospective multicenter pilot study of the safety and efficacy of microfocused ultrasound with visualization for improving lines and wrinkles of the decollete. Clinical Trial

Multicenter Study

Research Support, Non-U.S. Gov't. *Dermatol Surg*. Mar 2015;41(3):327-35. doi:<https://dx.doi.org/10.1097/DSS.0000000000000322>

47. Lin FG. Nonsurgical Treatment of Postpartum Lower Abdominal Skin and Soft-Tissue Laxity Using Microfocused Ultrasound With Visualization. *Dermatol Surg*. 12 2020;46(12):1683-1690. doi:<https://dx.doi.org/10.1097/DSS.0000000000002576>

48. Gold MH, Sensing W, Biron J. Use of micro-focused ultrasound with visualization to lift and tighten lax knee skin (1.). Clinical Trial

Research Support, Non-U.S. Gov't. *J Cosmet Laser Ther*. Oct 2014;16(5):225-9. doi:<https://dx.doi.org/10.3109/14764172.2014.949273>
